# Identification of Lipid Markers of *Plasmopara viticola* Infection in Grapevine Using a Non-targeted Metabolomic Approach

**DOI:** 10.3389/fpls.2018.00360

**Published:** 2018-03-21

**Authors:** Lise Negrel, David Halter, Sabine Wiedemann-Merdinoglu, Camille Rustenholz, Didier Merdinoglu, Philippe Hugueney, Raymonde Baltenweck

**Affiliations:** SVQV, Institut National de la Recherche Agronomique, Université de Strasbourg, Colmar, France

**Keywords:** grapevine, downy mildew, metabolomics, lipids, biomarkers

## Abstract

The Oomycete *Plasmopara viticola* is responsible for downy mildew, which is one of the most damaging grapevine diseases. Due to the strictly biotrophic way of life of *P. viticola*, its metabolome is relatively poorly characterized. In this work, we have used a mass spectrometry-based non-targeted metabolomic approach to identify potential *Plasmopara*-specific metabolites. This has led to the characterization and structural elucidation of compounds belonging to three families of atypical lipids, which are not detected in healthy grapevine tissues. These lipids include ceramides and derivatives of arachidonic and eicosapentaenoic acid, most of which had not been previously described in Oomycetes. Furthermore, we show that these lipids can be detected in *Plasmopara*-infected tissues at very early stages of the infection process, long before the appearance the first visible symptoms of the disease. Therefore, the potential use of these specific lipids as markers to monitor the development of *P. viticola* is discussed.

## Introduction

*Plasmopara viticola* is an obligate biotrophic Oomycete responsible for downy mildew, which is one of the most damaging grapevine diseases. As downy mildew outbreaks may result in significant economic losses, control of the disease is most often achieved by fungicide treatments, whose impacts on environment and health raise more and more concerns. The relationships between grapevine and *P. viticola* have therefore been subjected to thorough investigations in order to better understand the infection process and to identify potential targets for alternative control strategies (Gessler et al., 2011). Grapevine responses to downy mildew infection have been characterized in both compatible and incompatible situations, through the study of susceptible *Vitis vinifera* cultivars and resistant species such as *Vitis riparia, V. rupestris*, or *Muscadinia rotundifolia*. Transcriptomic analyses have shown that downy mildew infection induces a strong and rapid transcriptional reprogramming in host tissues, including the induction of pathogenesis-related proteins and enzymes required for the synthesis of phenylpropanoid-derived compounds (Kortekamp, 2006; Polesani et al., 2008, 2010; Wu et al., 2010; Gessler et al., 2011; Legay et al., 2011; Lenzi et al., 2016). Similarly, metabolomic profiling experiments have revealed profound changes in grapevine tissues upon downy mildew infection, which affect both the primary and the secondary metabolism (Figueiredo et al., 2008; Ali et al., 2009; Buonassisi et al., 2017). One of the most prominent metabolic change is the biosynthesis of large amounts of stilbene phytoalexins in both compatible and incompatible interactions (Pezet et al., 2003; Alonso-Villaverde et al., 2011), specific patterns of stilbene accumulation being associated with increased resistance to the pathogen (Malacarne et al., 2011; Duan et al., 2015; Chitarrini et al., 2017). However, metabolomic analyses of grapevine-downy mildew interactions have mostly resulted in the characterization of metabolites from grapevine, the metabolome of *P. viticola* being paradoxically relatively poorly characterized. This is probably partly due to the strictly biotrophic way of life of *P. viticola*, which thus cannot be obtained in pure culture, making the characterization of its metabolome difficult.

In this work, we have used a high-resolution mass spectrometry-based non-targeted metabolomic approach to characterize metabolites differentially accumulated in grapevine and in *P. viticola*. The rationale behind this strategy was to look for prominent ions present in *P. viticola* and absent in grapevine healthy tissues, which may be derived from *Plasmopara*-specific molecules. This has led to the characterization and structural elucidation of compounds belonging to three families of atypical lipids, which are not detected in healthy grapevine tissues. Furthermore, we show that these lipids can be detected in *Plasmopara*-infected tissues from very early stages of the infection process. Therefore, the potential use of these specific lipids as markers to monitor the development of *P. viticola* is discussed.

## Materials and methods

### Chemicals and standards

LC-MS grade methanol and chloroform were from Roth Sochiel (Lauterbourg, France), water was provided by a Millipore water purification system. Arachidonic acid, eicosapentaenoic acid and N-Arachidoyl-D-sphingosine Cer(d18:1/20:0) were purchased from Sigma–Aldrich (Saint-Quentin Fallavier, France). N-Hexadecanoyl-D-erythro-C16-sphingosine Cer(d16:1/16:0) was from Abcam (Paris, France). Triarachidonoyl-glycerol and trieicosapentaenoyl-glycerol were purchased from Larodan Fine Chemicals (Malmö, Sweden). AG, DAG, EPG, and DEPG were obtained from AA and EPA by ZnCl_2_-catalyzed esterification of glycerol (Spring and Haas, 2004; Mostafa et al., 2013).

### Plant material and downy mildew inoculation

The isolate SC of *P. viticola* used in this work and the leaf disc bioassay were described previously (Peressotti et al., 2010). Inoculum (sporangia) was prepared from infected leaves of *V. vinifera* cv Muscat Ottonel treated with Topsin 70 WG fungicide (50 mg/L) (Nisso Chemical, Germany), in order to avoid fungal contamination (Peressotti et al., 2010). Before inoculation leaves were surface-sterilized with bleach (2% active chlorine) for 2 min, followed by three washes with sterile water. In this work, the leaf disc bioassay was conducted with the following grapevine varieties and species: *V. vinifera* cv Cabernet-Sauvignon, cv Syrah, cv Cinsault, *V. rupestris, V. riparia*, and *V. rotundifolia*, using 15 leaf discs for each variety. Kinetic analysis of *Plasmopara*-specific lipids accumulation was performed on green cuttings of *V. vinifera* cv Syrah, the hybrid variety Bianca and *V. riparia*, grown in a greenhouse. The sixth leaf counted from the apex of 3.5 months old plants, were harvested, surface-sterilized, and washed with sterile water. Leaves were infected by flotation of their abaxial surface on a suspension of sporangia (10^5^ sporangia mL^−1^) for 5 h. Control leaves were floated on sterile water. Three leaves were used for each treatment. The leaves were then transferred to wet paper with the abaxial surface up and closed in transparent plastic bags. Leaves were stored in a growth chamber at 21°C, with 16 h of light per day. 0, 24, 48, 72 h, and 6 days following inoculation, discs (diameter 1.5 cm) were punched from infected and control leaves, weighed, placed in a 2 mL tube, frozen into liquid nitrogen and stored at −80°C until analysis.

### Preparation of sporangia extracts from *P. viticola*

The abaxial faces of the leaves of 2 month-old Muscat Ottonel plants were sprayed with a suspension of sporangia (10^5^ sporangia mL^−1^ in water). Mock-inoculated leaves were sprayed with sterile water. Three inoculated and three control plants were then placed separately in growth chambers at 21°C, with 16 h of light per day. Seven days later, two leaves per plant were carefully cut and randomly distributed in three groups. Sporulating leaves were shaken in 35 mL ultrapure water to recover sporangia. The three suspensions of sporangia were adjusted to 10^5^ sporangia mL^−1^ and 30 mL of each suspension was centrifuged at 30,000 g for 20 min. Pellets of sporangia were extracted with 2 mL of MeOH/CHCl_3_ (1/1, v/v), by sonicating for 15 min in an ultrasound bath. Two mL of ultrapure water was added to allow phase separation. After centrifugation at 15,000 g for 10 min, the chloroform phase was concentrated to 100 μL and diluted with 100 μL of MeOH. Final extracts corresponded to 1.5.10^7^ sporangia mL^−1^. Mock inoculated leaves were extracted as described below.

### Metabolite extraction from grapevine leaves

Leaf discs were freeze-dried and weighed. After grinding the discs using a bead mill (TissueLyser II, Qiagen, Courtaboeuf, France), metabolites were extracted with MeOH/CHCl_3_ (1/1, v/v) using 15 μL per mg dry weight. The suspension of leaf powder in MeOH/CHCl_3_ was then sonicated for 15 min in an ultrasound bath. Two milliliters of ultrapure water was added to allow phase separation. After centrifugation at 15,000 g for 10 min, 100 μL of the chloroform phase was recovered, diluted with 100 μL of MeOH and analyzed by UHPLC-MS.

### UHPLC-HRMS

Analyses were performed using a Dionex Ultimate 3000 UHPLC system (Thermo Fisher Scientific, San Jose, USA). The chromatographic separation was performed on a Nucleodur C18 HTec column (50 × 2 mm, 1.8 μm particle size; Macherey-Nagel, Düren, Germany) maintained at 20°C. The mobile phase consisted of methanol with formic acid (0.1%, v/v) in isocratic elution at a flow rate of 0.30 mL/min. The sample volume injected was 1 μL. The UHPLC system was coupled to an Exactive Orbitrap mass spectrometer (Thermo Fischer Scientific) equipped with an atmospheric pressure chemical ionization (APCI) source operating in positive mode. Parameters were set at 300°C for ion transfer capillary temperature and the corona discharge current was set at 5 μA. Nebulization with nitrogen sheath gas and auxiliary gas were maintained at 15 and 10 arbitrary units, respectively, and the nebulizer temperature was maintained at 400°C. The spectra were acquired within the *m/z* mass range of 100–1,200 atomic mass units (amu), using a resolution of 50,000 at *m/z* 200 amu. The system was calibrated internally using dibutylphthalate as lock mass at *m/z* 279.1591, giving a mass accuracy lower than 1 ppm. The instruments were controlled using the Xcalibur software and data was processed using the XCMS software package (Smith et al., 2006). Raw data were converted to the mzXML format using MSconvert before analysis. Settings of the xcmsSet function of XCMS were as follows: method = “centWave”, ppm = 2, noise = 50,000, mzdiff = 0.001, prefilter = c(5,15,000), snthresh = 6, peakwidth = c(6,35). Peaks were aligned using the obiwarp function using the followings settings of the function group.density: bw = 10, mzwid = 0.0025. This allowed the alignment of 2,156 peaks in the positive mode. Ion identifiers were generated automatically by XCMS as MxxxTyyy, where xxx is the *m/z* and yyy the retention time in seconds. Selection of major differential ions (peak area > 10^6^, fold > 15) between sporangia and leaf extracts led to a final set of 123 major ions. The integration of each peak was checked manually before validation. *T*-tests were performed in R with the *t.test* function. Differential ions with *p* ≤ 0.05 were considered for further characterization. The exact *m/z* and retention time of each metabolite were used for targeted metabolomic analyses using the Excalibur software. For kinetic analysis of *P. viticola* development, seven major *Plasmopara*-specific lipids [AA, DAG, EPA, DEPG, TEPG, Cer(d16:1/18:0), Cer(d16:1/20:0)] were selected.

## Results

### Characterization of candidate *Plasmopara*-specific metabolites using non-targeted metabolomics

As an obligate biotrophic pathogen, *P. viticola* cannot be grown in pure culture. However, sporangia can easily be collected from infected grapevine leaves. In order to characterize potential *Plasmopara*-specific metabolites, we compared extracts of sporangia and uninfected grapevine leaves using ultra high-performance liquid chromatography coupled to high-resolution mass spectrometry (UHPLC-HRMS). This differential metabolomic approach has led to the selection of a set of 123 major ions (peak area > 10^6^, *p* ≤ 0.05), which are at least 15 times more abundant in sporangia extracts than in leaf extracts (Supplementary Table 1). The level of contamination of sporangia extracts by leaf-derived material was estimated by quantifying chlorophyll *a*. Chlorophyll *a* content in sporangia extracts (1.7.10^4^ ± 1.2.10^4^ arbitrary units) was 4,000 times lower than in leaf extracts (6.8.10^7^ ± 4.1.10^7^ arbitrary units), indicating very low levels of contamination. Submission of these 123 ions to the Metlin database (Smith et al., 2005) led to the putative identification of two compounds of particular interest. Indeed, one of the proposed structures for compounds giving the ion n°10 (M303T44) with the mass-to-charge ratio (*m/z*) 303.2316 was eicosapentaenoic acid (EPA). Similarly, ion n°12 (M305T48) with *m/z* 305.2473 was potentially associated to arachidonic acid (AA) (Table 1). Both EPA and AA had previously been identified in mycelial extracts of *Phytophthora infestans*, another phytopathogenic Oomycete causing the late blight of potato (Bostock et al., 1981). Commercial EPA and AA standards were therefore analyzed by UHPLC-HRMS using the same conditions as those used for *P. viticola* extracts. The retention times (RTs) and fragment patterns of the standards were identical to those of M303T44 (n°10) and M305T48 (n°12) (Supplementary Figure 1), confirming their identities as EPA and AA, respectively.

**Table 1:** *Plasmopara*-specific ions identified by non-targeted metabolomics, which are referred to in the text.

**Ion n°**	**Identifier**	**m/z**	**RT(s)**	**Ion formula**	**Identification**
**10**	**M303T44**	**303.2316**	**43.87**	**C**_**20**_**H**_**30**_**O**_**2**_	**eicosapentaenoic acid (EPA)**
**5**	**M377T39**	**377.2683**	**38.81**	**C**_**23**_**H**_**37**_**O**_**4**_	**eicosapentaenoyl-glycerol (EPG)**
**4**	M359T39	359.2577	38.81	**C**_**23**_**H**_**35**_**O**_**3**_	fragment from EPG [M-H_2_O+H]^+^
**16**	**M661T85**	**661.4825**	**84.66**	**C**_**43**_**H**_**65**_**O**_**5**_	**dieicosapentaenoyl-glycerol (DEPG)**
**117**	**M946T474**	**945.6964**	**473.78**	**C**_**63**_**H**_**93**_**O**_**6**_	**trieicosapentaenoyl-glycerol (TEPG)**
**12**	**M305T48**	**305.2474**	**47.97**	**C**_**20**_**H**_**32**_**O**_**2**_	**arachidonic acid (AA)**
**8**	**M379T42**	**379.2839**	**41.59**	**C**_**23**_**H**_**39**_**O**_**4**_	**arachidonoyl-glycerol (AG)**
**30**	**M666T124**	**665.5134**	**123.80**	**C**_**43**_**H**_**69**_**O**_**5**_	**diarachidonoyl-glycerol (DAG)**
**19**	**M492T96**	**492.4773**	**96.14**	**C**_**32**_**H**_**64**_**NO**_**3**_	**Cer(d16:1/16:0)**
**40**	**M539T125**	**538.5189**	**125.15**	**C**_**34**_**H**_**68**_**NO**_**3**_	***Cer(d16:1/18:0)***
**33**	M236T125	236.2371	125.15	C_16_H_30_N	*fragment N from Cer(d16:1/18:0)*
**34**	M254T125	254.2477	125.15	C_16_H_32_NO	*fragment N′ from Cer(d16:1/18:0)*
**37**	M521T125	520.5084	125.15	C_34_H_66_NO_2_	*fragment [M-H_2_O+H]^+^ from Cer(d16:1/18:O)*
**42**	M356T126	356.3156	125.61	C_21_H_42_NO_3_	*fragment C from Cer(d16:1/18:0)*
**43**	M284T126	284.2945	126.06	C_18_H_38_NO	*fragment A from Cer(d16:1/18:0)*
**73**	**M567T166**	**566.5502**	**165.80**	**C**_**36**_**H**_**72**_**NO**_**3**_	***Cer(d16:1/20:0)***
**66**	M236T165	236.2371	164.83	C_16_H_30_N	*fragment N from Cer(d16:1/20:0)*
**67**	M254T165	254.2476	164.83	C_16_H_32_NO	*fragment N′ from Cer(d16:1/20:0)*
**71**	M549T166	548.5399	165.69	C_36_H_70_NO_2_	*fragment [M-H_2_O+H]^+^ from Cer(d16:1/20:0)*
**97**	**M595T223**	**594.5818**	**223.18**	**C**_**38**_**H**_**76**_**NO**_**3**_	***Cer(d16:1/22:0)***
**93**	M236T223	236.2372	223.18	C_16_H_30_N	*fragment N from Cer(d16:1/22:0)*
**94**	M412T223	412.3784	223.18	C_25_H_50_NO_3_	*fragment C from Cer(d16:1/22:0)*
**95**	M577T223	576.5713	223.18	C_38_H_74_NO_2_	*fragment [M-H_2_O+H]^+^ from Cer(d16:1/22:0)*
**13**	M281T50	281.2473	50.26	C_18_H_32_O_2_	*C_18:2_ acid*
**46**	**M642T142**	**641.5134**	**141.80**	**C**_**41**_**H**_**69**_**O**_**5**_	***diglyceride 20:5/18:1***

*Major compounds are indicated in bold. Identifications confirmed using the corresponding standards are marked by a gray background. Ions are numbered according to their retention time and are organized as derivatives of EPA, AA, and ceramides, indicated in green, blue, and red, respectively. Putative identifications are indicated with italics. For all ions, the identifier, the m/z, and retention time (RT) are indicated. For fragment nomenclature, see Supplementary Figure 3. The original list of ions identified by non-targeted metabolomics on one experiment including three biological replicates of extracts of P. viticola sporangia and of mock-inoculated leaves is presented in Supplementary Table 1 and the full list of 51 ions identified in this work is presented in Supplementary Table 2*.

### Structural elucidation of EPA-containing lipids

**Figure 1:**
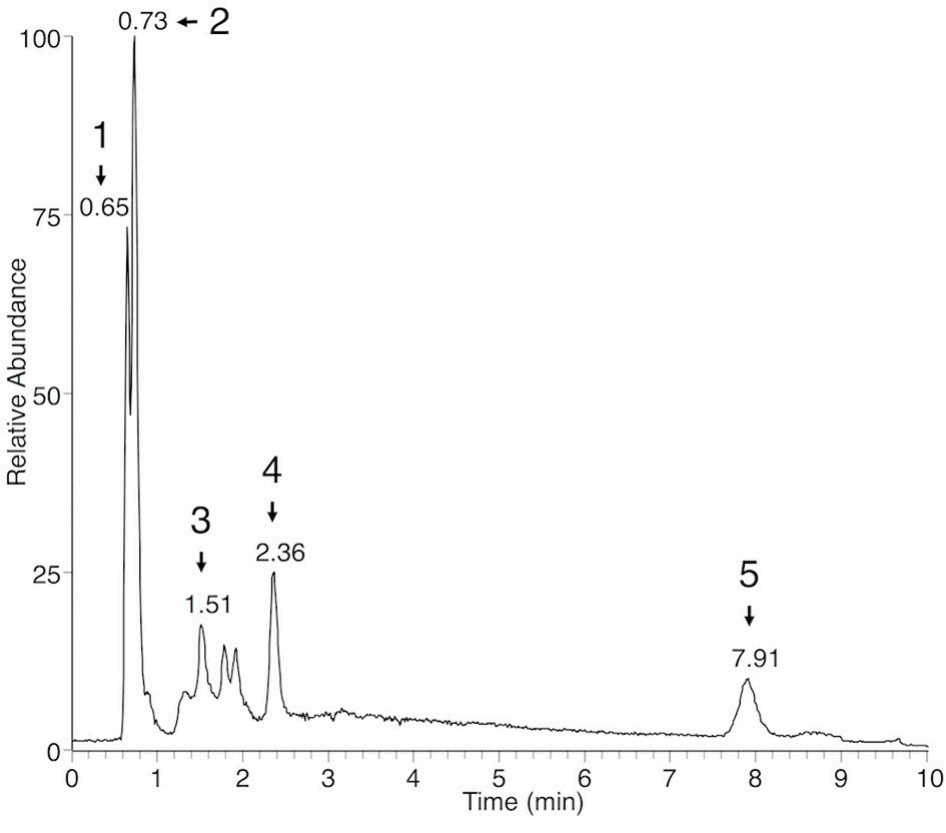
Extracted ion chromatogram of sporangia extracts showing putative EPA-containing lipids. EIC of sporangia extracts for *m/z* 285.2212 (±2 ppm), a characteristic ion of EPA, is presented. Peaks corresponding to EPA-containing ions (Table 1) are numbered from 1 to 5 and retention times are indicated. Peak 1: M377T39 = EPG; peak 2: M303T44 = EPA; peak 3: M661T85 = DEPG; peak 4: M642T142 = diglyceride 20:5/18:1; peak 5: M946T474 = TEPG. Identity of EPA, EPG, DEPG, and TEPG have been confirmed by using the corresponding standards.

In both the *P. viticola* extracts and the commercial standard, in source fragmentation of EPA produced a characteristic ion with *m/z 285*.2212 (C_20_H_29_O) corresponding to a loss of water. Extracted ion chromatogram (EIC) for *m/z* 285.2212 showed that other metabolites gave rise to this particular ion in *P. viticola* sporangia extracts (Figure 1), indicating that these molecules may contain EPA. A more detailed analysis of the mass spectra of some of these metabolites showed that they were actually present in our list of major *Plasmopara*-specific ions (Figure 1, Table 1). This prompted us to characterize these putative EPA-containing compounds further. The mass and the formula of some of these compounds indicated that they may correspond to glycerides, constituted by glycerol (C_3_H_8_O_3_) partially or totally esterified by EPA. For example, the ion M377T39 (n°5) with *m/z* 377.2683, corresponding to the formula C_23_H_37_O_4_ (average mass error, AME 0.8 ppm), was putatively identified as eicosapentaenoyl-glycerol (EPG). Indeed, it gave rise to a first dehydration fragment with *m/z* 359.2579 (C_23_H_35_O_3_), as well as to the characteristic dehydration fragment of EPA (*m/z* 285.2210). The first fragment corresponded to the ion M359T39 (n°4). The same reasoning could be applied to the ions M661T85 (n°16) and M946T474 (n°117), which where therefore putatively identified as dieicosapentaenoyl-glycerol (DEPG) and trieicosapentaenoyl-glycerol (TEPG), respectively. The identification of EPA glycerides was confirmed by comparing their characteristics to those of the corresponding synthetic standards (see below). Altogether, EPA and EPA glycerides give rise to 15 of 123 the major *Plasmopara*-specific ions selected in Supplementary Table 2.

### Structural elucidation of AA-containing lipids

In source fragmentation of the AA standard gave rise to a characteristic ion with *m/z* 287.2369 (C_20_H_31_O, AME 0.1 ppm) corresponding to a loss of water. Using the same strategy as with EPA, EIC for *m/z* 287.2369 revealed several potential AA-containing molecules in *P. viticola* sporangia extracts (Supplementary Figure 2), some of them being listed among the *Plasmopara*-specific compounds (Table 1). Similarly, the formula and the fragmentation pattern of some of them indicate that they may be AA glycerides. For example, the ion M379T42 (n°8, Table 1) with *m/z* 379.2839 (C_23_H_39_O_4_, AME 1 ppm) could be putatively identified as arachidonoyl-glycerol (AG), as it produced a dehydration product with *m/z* 361.2736 (C_23_H_37_O_3_, AME 0.1 ppm) together with the characteristic dehydration ion of AA (*m/z* 287.2369, C_20_H_31_O). Applying the same reasoning, the ion M666T124 (n°30) was putatively identified as diarachidonoyl-glycerol (DAG). As TEPG was abundant in *P. viticola* sporangia, we logically looked for triarachidonoyl-glycerol (TAG), although no ion potentially derived from this compound was found in Table 1. TAG was indeed detected, albeit with a retention time of 21.81 min, exceeding the 10 min limit originally set for the XCMS analysis, and therefore explaining its absence in Table 1. In order to confirm the identity of the fatty acid glycerides detected in *P. viticola* extracts, the corresponding standards were synthesized by ZnCl_2_-catalyzed esterification of glycerol (Mostafa et al., 2013). Using EPA and AA, mixtures of glycerides of the corresponding fatty acids were obtained and analyzed by UHPLC-HRMS using the same conditions as those used for sporangia extracts. The synthetic AG, DAG, EPG, and DEPG exhibited the same RT and fragment patterns as the corresponding compounds detected *P. viticola* extracts, therefore confirming their identity. The identities of the triglycerides were confirmed using the corresponding commercial TAG and TEPG standards.

### Characterization and structural elucidation of further *Plasmopara*-specific lipids

**Figure 2:**
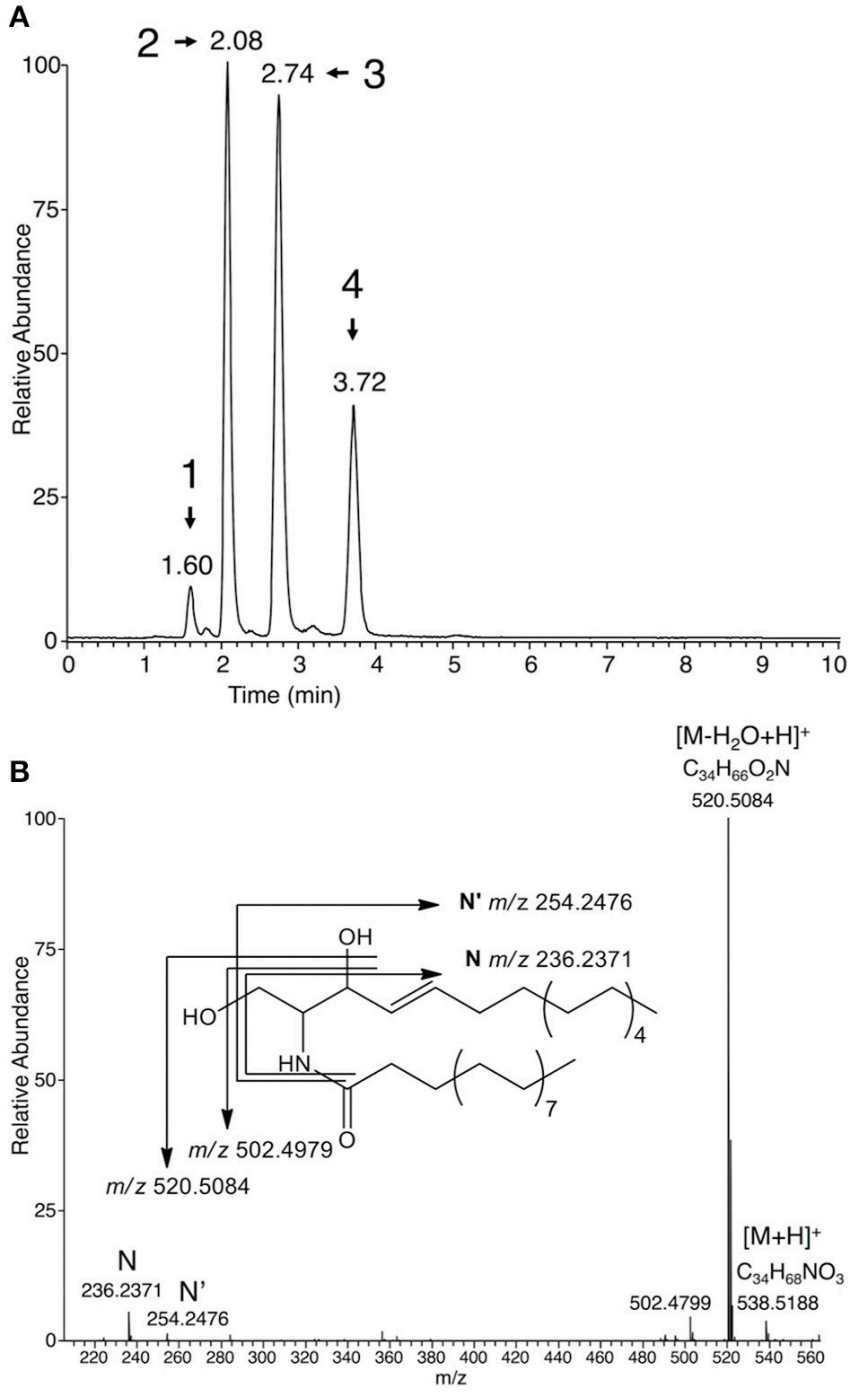
Characterization of ceramides in sporangia extracts. **(A)** EIC of sporangia extracts for *m/z* 236.2371 (±2 ppm), a characteristic ion of C16:1 ceramides. Peaks corresponding to *Plasmopara*-specific ions (Table 1) are numbered from 1 to 4 and retention times are indicated. Peak 1: M492T96 = Cer(d16:1/16:0); peak 2: M521T125 = Cer(d16:1/18:0); peak 3: M549T166 = Cer(d16:1/20:0); peak 4: Cer(d16:1/22:0). Identity of Cer(d16:1/16:0) was confirmed by using the corresponding standard. **(B)** Mass spectrum of putative Cer(d16:1/18:0) (M521T125) from *P. viticola*. The fragmentation giving rise to the main fragments is indicated. A detailed fragmentation is given in Supplementary Figure 3.

Among putative *Plasmopara*-specific metabolites, the ion with *m/z* 236.2371 (putative formula C_16_H_30_N) appeared three times at different RTs (ions n°33, 66, and 93, at RT 125, 165, and 223 s, respectively; Table 1). For all RTs, this ion seemed to belong to a group of ions corresponding to fragments of larger metabolites. Indeed, EIC for *m/z* 236.2371 revealed four different peaks at RTs 96, 125, 165, and 223 s (Figure 2A). Analysis of the major ions present at these RTs revealed four compounds, which are also present in Table 1, namely M492T96, M521T125, M549T166, M577T223 (n°19, n°37, n°71, n°95). Each of these ions exhibited a mass difference of 28.031 atomic mass units (amu) with the following one, this mass increase being compatible with their belonging to a family of compounds that differentiate themselves by an increasing number of ethylene (C_2_H_4_) groups. Therefore, the putative formula of M492T96 (n°19) was C_32_H_62_O_2_N, and the following metabolites corresponded to the putative formulas C_34_H_66_O_2_N, C_36_H_70_O_2_N, C_38_H_74_O_2_N, respectively. Further analysis of the mass spectra revealed that, alongside the major ions listed above, we could detect minor ions corresponding to the same formula with either a loss of water or one additional water molecule (H_2_O). The latter ions corresponded to *m/z* 510.4877, 538.5189, 566.5502, and 594.5818, respectively. Search of the Metlin database for compounds possibly corresponding to these ions (with 1 ppm AME) retrieved candidates belonging to the ceramide family, composed of a C16:1 or C18:1 sphingosine, linked to a C14, C16, C18, C20, or C22 saturated fatty acid (Supplementary Table 3). In order to validate these potential candidates, a commercial standard of the ceramide N-arachidoyl-D-sphingosine Cer(d18:1/20:0), which may correspond to the ion M577T223 (n°95), was analyzed by UHPLC-HRMS using the same conditions as those used for *P. viticola* extracts. Its mass spectrum showed a [M+H]^+^ pseudo molecular ion with *m/z* 594.5814 corresponding to C_38_H_76_O_3_N, together with several fragments with *m/z* 264.2683 (C_18_H_36_N), *m/z* 282.2791 (C_18_H_38_ON) and *m/z* 576.5713 (C_38_H_74_O_2_N), which was the major fragment. Ions coming from the fatty acid part were detected too (*m/z* 312.3258, C_20_H_42_ON and *m/z* 354.3363, C_22_H_44_O_2_N), in agreement with the fragmentation of ceramides described previously in several studies (Levery et al., 2000; Hsu and Turk, 2002; Murphy, 2015) and obtained with this standard (Supplementary Table 3). However, neither its RT nor its fragmentation corresponded to any of the ions found in *P. viticola*, excluding the hypothesis that some of these ions may be derived from C18:1 sphingosines.

**Figure 3:**
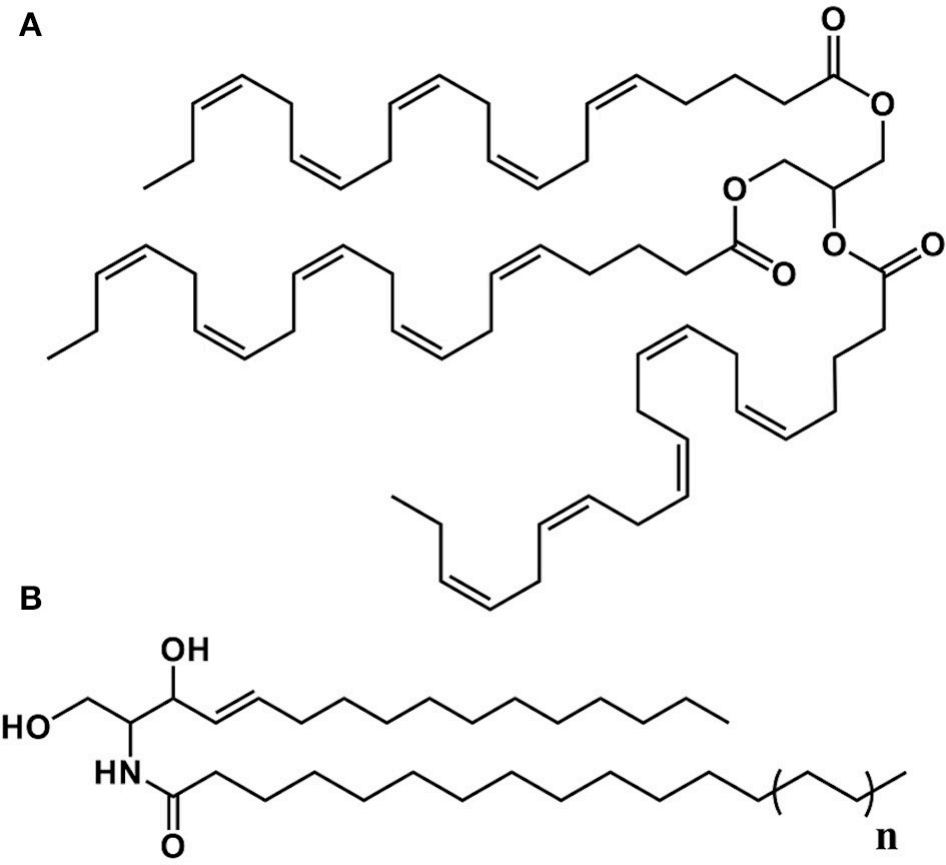
Structure of some of the lipids characterized in *P. viticola*. **(A)** Trieicosapentaenoyl-glycerol (TEPG). **(B)** *n* = 1: Cer(d16:1/16:0); *n* = 2: Cer(d16:1/18:0); *n* = 3: Cer(d16:1/20:0); *n* = 4: Cer(d16:1/22:0).

A second set of candidates retrieved from the Metlin database contained C16:1 sphingosine. Indeed, the four compounds present in *P. viticola* extracts shared the same specific fragments with *m/z* 236.2371 (C_16_H_30_N) and 254.2476 (C_16_H_32_ON) (Figure 2B). The formulas of these fragments could be compatible with their being derived from a C16:1 sphingosine (Lhomme et al., 1990; Pivot et al., 1994). Therefore, a commercial standard of the ceramide Cer(d16:1/16:0) was analyzed by UHPLC-HRMS. Both its RT and its fragmentation pattern corresponded to that of the ion M492RT96 (n°19) found in *P. viticola* (Supplementary Table 3). Furthermore, M492RT96 and Cer(d16:1/16:0) exhibited the same RT in three different chromatographic conditions (data not shown), showing that the ion M492RT96 (n°19) is indeed derived from Cer(d16:1/16:0) present in *P. viticola* extracts (Figures 2A, 3). The ions M492T96, M521T125, M549T166, and M577T223 (n°19, n°37, n°71, n°95) all share common fragments with *m/z* 236.2373 and 254.2476, corresponding to C_16_H_30_N et C_16_H_32_ON, respectively, which are derived from the C16:1 sphingosine. As for the commercial standards Cer(d16:1/16:0) and Cer(d18:1/20:0), three ions coming from the fatty acid part are detected for all four ceramides (Supplementary Table 3). For Cer(d16:1/18:0) (n°37), these characteristic fragments termed A, B, and C are indicated in Supplementary Figure 3. Therefore, the ions M521T125, M549T166, and M577T223 (n°37, n°71, n°95) are very likely to be derived from a family or ceramides with a C16:1 sphingosyl group bound to a saturated fatty acid with 18–22 carbon atoms, namely Cer(d16:1/18:0), Cer(d16:1/20:0), and Cer(d16:1/22:0), respectively (Figures 2A, 3). Detailed analysis of the fragments derived from these compounds is presented in Supplementary Table 3.

Altogether, we could propose an identification for 51 (Supplementary Table 2) of the final set of 123 major ions, which are at least 15 times more abundant in sporangia extracts than in leaf extracts (with *p* ≤ 0.05, Supplementary Table 1).

### *Plasmopara*-specific lipids can be used for phenotyping resistance to downy mildew

**Figure 4:**
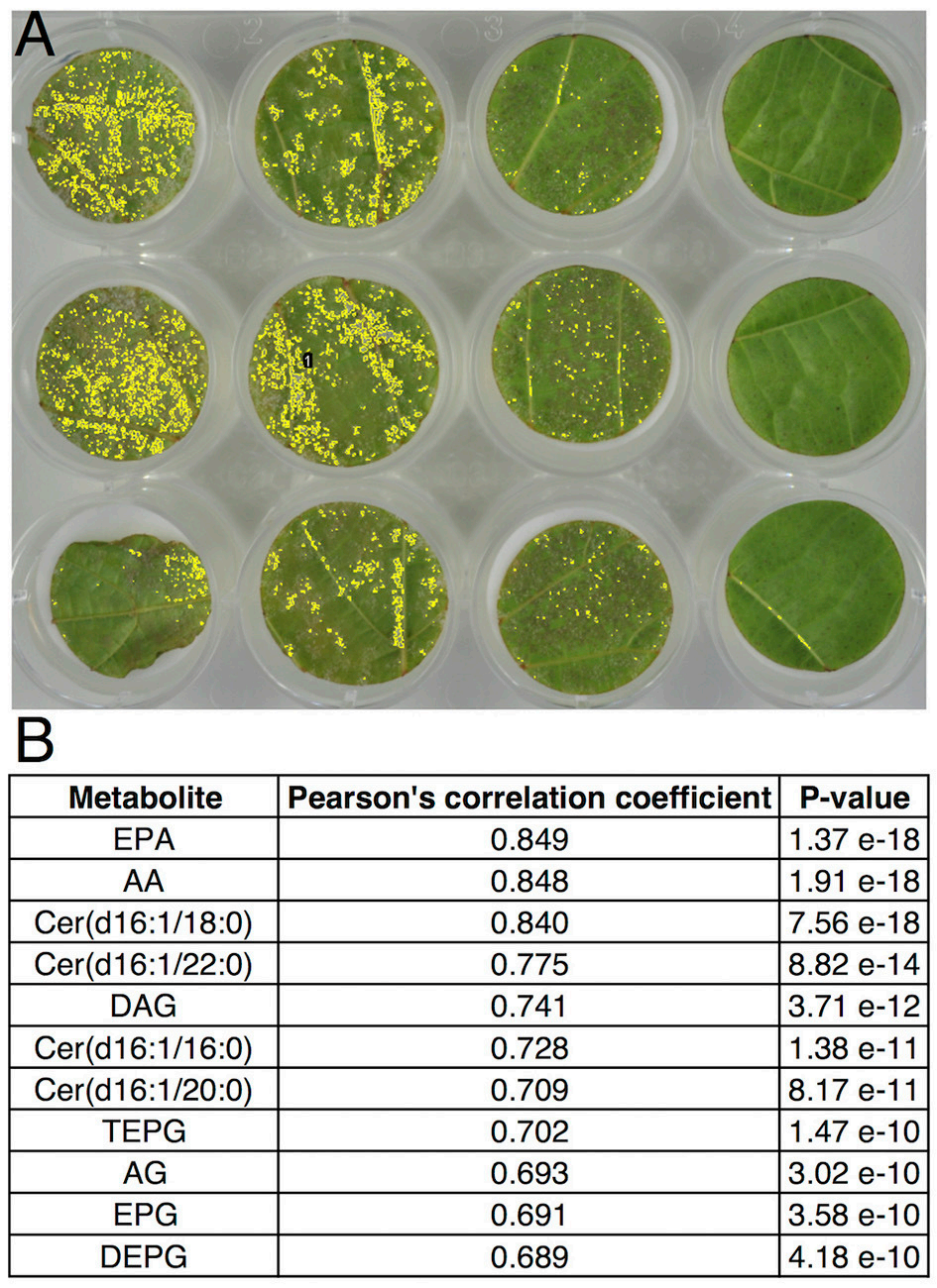
Correlation between image analysis-based quantification of sporulation and quantification of lipid markers of *P. viticola*. Leaf disc bioassay was performed with Cabernet-Sauvignon, Syrah, Cinsault, *V. rupestris, V. riparia*, and *V. rotundifolia*, using 15 leaf discs for each variety. **(A)** Typical quantification of sporulating area 6 days post inoculation using image analysis (Peressotti et al., 2011). **(B)** Correlation and associated *p*-value of individual lipid marker quantification with sporulating area, using Pearson's correlation test.

The accurate quantification of pathogen development is important for a better understanding of plant-pathogen interactions. In the case of *P. viticola*, methods based on cell counting or image analysis are routinely used to quantify sporulation, especially as a phenotyping tool to characterize resistance sources in the *Vitis* genus (Peressotti et al., 2011; Divilov et al., 2017). Our differential metabolomic approach has led to the characterization of lipids, which are abundant in the sporangia of *P. viticola* and not detected in healthy grapevine leaf tissues. In order to evaluate the possibility to use these compounds for phenotyping resistance to *P. viticola*, analysis of lipid markers was compared to image analysis for quantifying sporulation in a leaf disc assay routinely used to assess resistance to *P. viticola* (Figure 4A; Peressotti et al., 2011). Correlations between sporulation and quantification of all individual lipid markers were very good (Figure 4B), all associated *p*-values being lower than 10^−9^. This demonstrates the specificity of the lipid markers and confirms that they are perfectly suited for phenotyping resistance to *P. viticola*. In particular, EPA and AA are giving the best correlation coefficients, meaning that they are very valuable markers for phenotyping resistance.

### Early detection and kinetic analysis of *Plasmopara*-specific lipids accumulation during downy mildew infection

**Figure 5:**
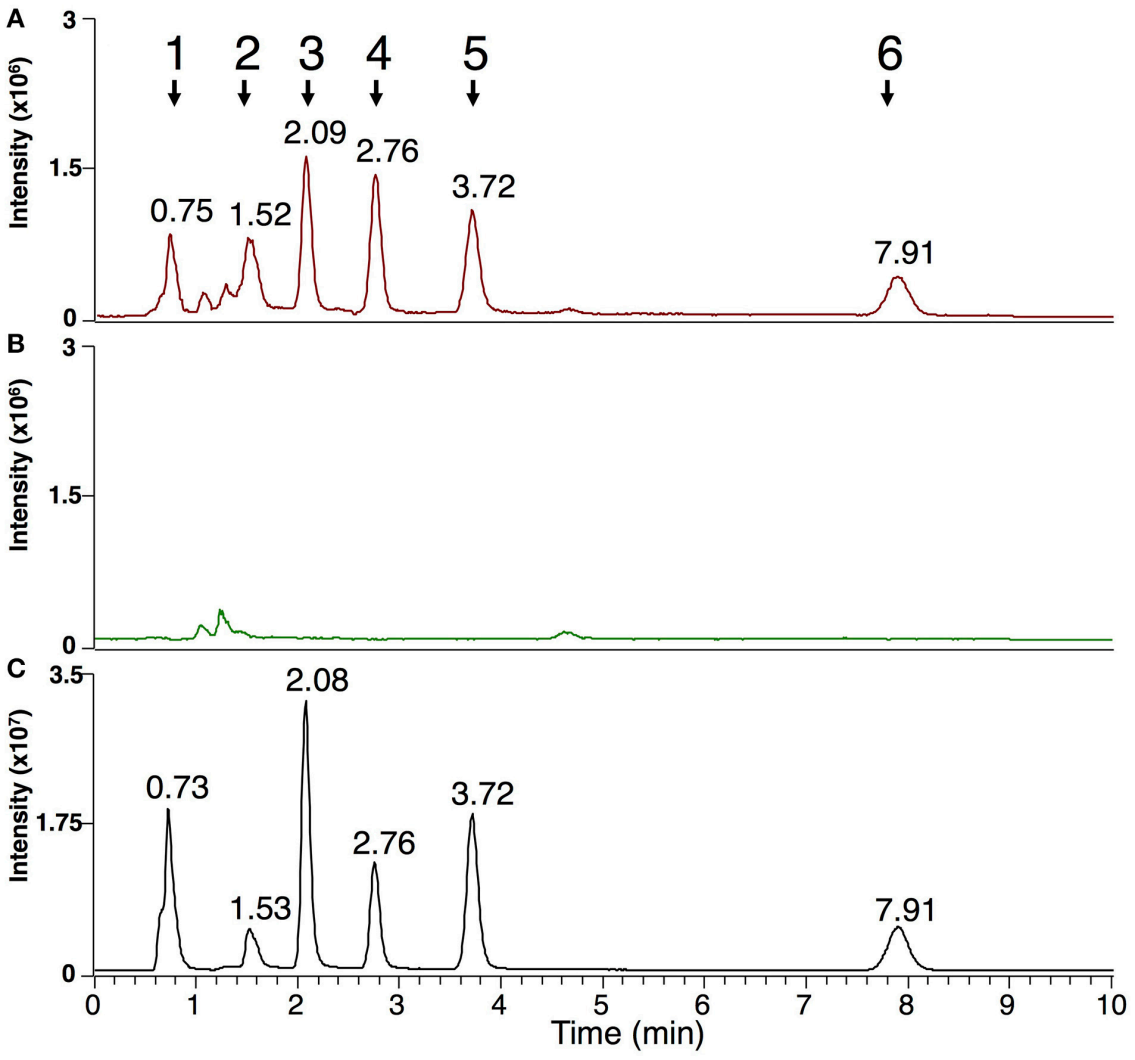
Markers of *P. viticola* in downy mildew-infected grapevine leaves before sporulation. Analysis of MeOH/CHCl_3_ extracts presenting the sum of the EIC for *m/z* of the characterized lipids of *P. viticola* (±2 ppm): 303.2316 + 305.2474 + 377.2683 + 379.2839 + 492.4773 + 520.5084 + 548.5399 + 576.5713 + 661.4825 + 665.5134 + 945.6964. **(A)** Typical pattern obtained from downy mildew-infected leaves (Muscat Ottonel) 4 days post infection; **(B)** control leaves; **(C)** sporangia of *P. viticola*. For easier comparison, infected **(A)** and control leaves **(B)** are presented at the same intensity scale (3.10^6^ arbitrary units). Peaks corresponding to lipids of *P. viticola* are numbered from 1 to 6 and the corresponding retention times are indicated. Peak 1: AA, EPA, AG, EPG; peak 2: DAG, DEPG, Cer(d16:1/16:0); peak 3: Cer(d16:1/18:0); peak 4: Cer(d16:1/20:0); peak 5: Cer(d16:1/22:0); peak 6: TEPG.

**Figure 6:**
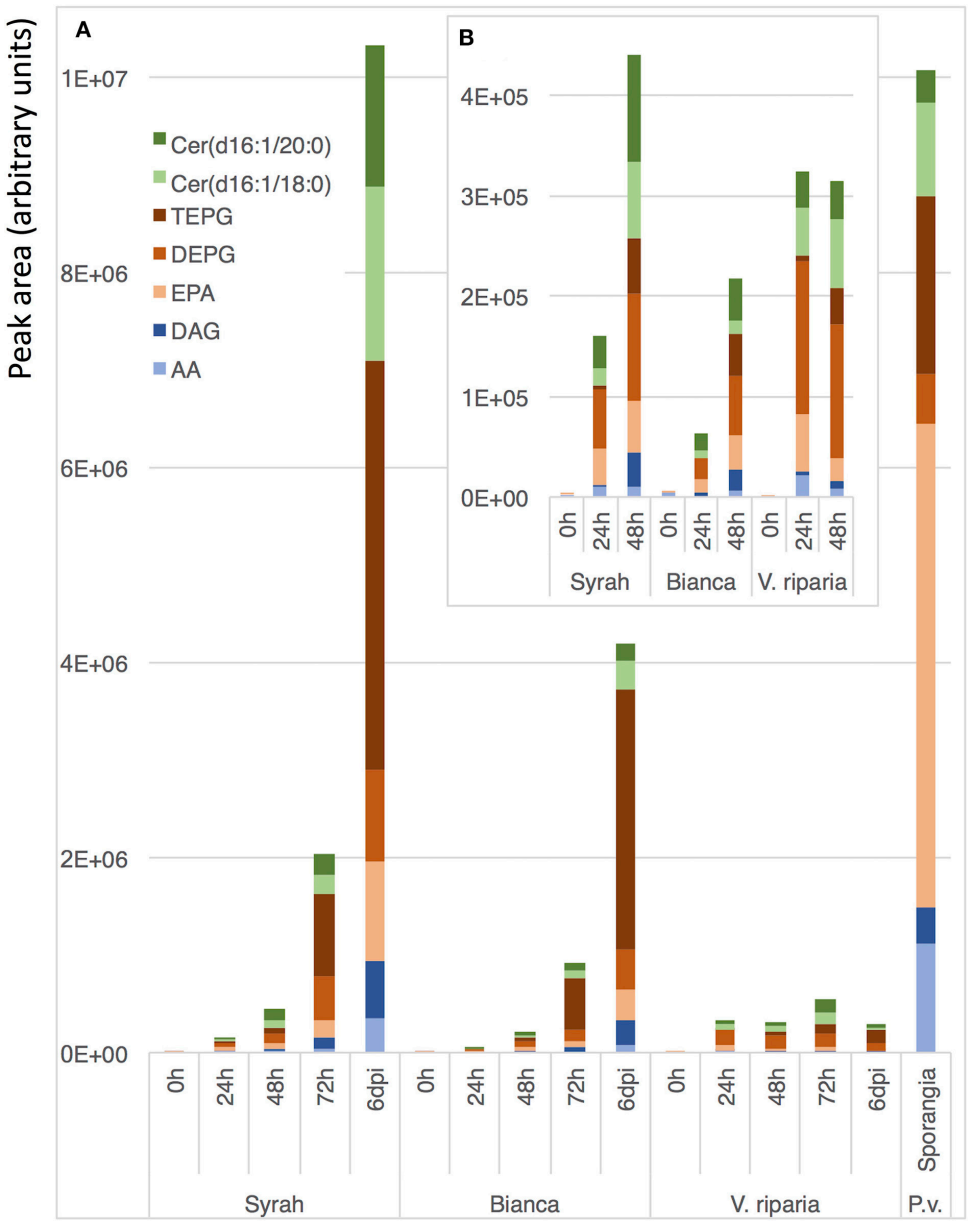
Kinetic analysis of *Plasmopara*-specific lipids accumulation. Genotypes with different susceptibility to downy mildew were used: Syrah (susceptible), Bianca (partially resistant) and *V. riparia* (resistant). **(A)** Quantification of the 7 major lipids 0, 24, 48, 72 h, and 6 days post inoculation (dpi) with *P. viticola*. As a comparison, a typical lipid profile of sporangia of *P. viticola* is indicated. **(B)** Enlargement for early infection times (0–48 h). Three biological replicates were analyzed for each condition and means are presented. A detailed analysis of *Plasmopara*-specific lipids in these samples is presented in Supplementary Figure 4 and the associated statistics are presented in Supplementary Table 4.

Beside end-point quantification of sporulation, an accurate monitoring of disease progression is important for precise description of disease susceptibility phenotypes. Therefore, we evaluated the possibility to use *Plasmopara*-specific lipids to quantify *P. viticola* in grapevine tissues before sporulation, i.e., as early and dynamic markers of downy mildew infection. Comparison of infected and mock-inoculated leaves showed that all ceramides and fatty acid derivatives described above were easily detected in the extract of infected leaves before sporulation, all these compounds being absent in control leaves (Figure 5). This prompted us to evaluate the potential of the *Plasmopara*-specific lipids to monitor the kinetic of *P. viticola* infection in three grapevines genotypes, which differ in their susceptibility to downy mildew: Syrah, Bianca, and *V. riparia*. Lipid analysis at different times post-inoculation allowed effective monitoring of disease development throughout the infection process, all lipids being efficiently detected even at early infection stages (24 h post-inoculation; Figure 6, Supplementary Figure 4, Supplementary Table 4). Different patterns and timings of *Plasmopara*-specific lipids accumulation were obtained in grapevine varieties with different susceptibility to downy mildew. However, patterns of *Plasmopara*-specific lipid accumulated in infected grapevine leaves differed significantly from that of isolated sporangia.

## Discussion

### *P. viticola* contains unusual lipids, which are not detected in healthy grapevine leaf tissues

Looking for potential markers of downy mildew infection using a non-targeted metabolomic approach, we could identify derivatives of *Plasmopara*-specific lipids, which include EPA and AA-containing lipids, in addition to ceramides. Both EPA and AA have previously been isolated from extracts of *P. infestans*, as elicitors of the accumulation of antimicrobial stress metabolites in potato tubers (Bostock et al., 1981). EPA and AA have been detected in a number of other Oomycetes (Gandhi and Weete, 1991; Spring and Haas, 2002; Pang et al., 2015), and more recently, the role of EPA and AA as microbial associated molecular patterns (MAMPs) has been extended to other plant-Oomycete interactions (Savchenko et al., 2010; Bostock et al., 2011). Due its remarkable abundance in *Plasmopara halstedii*, EPA has been proposed as a marker for downy mildew contamination in sunflower seeds (Spring and Haas, 2004). Similarly, fatty acids, including AA, have been used to detect *Phytophthora sojae* in soybean fields (Yousef et al., 2012). However, lipid analysis in Oomycetes has mostly targeted fatty acids released after total lipid hydrolysis and the information on complex lipids is very scarce. Here, we have used UHPLC-HRMS to directly characterize complex lipids without the modification usually needed for GC-MS analysis (hydrolysis, derivatization). In particular, optimized mild atmospheric pressure chemical ionization (APCI) has allowed the identification of ions corresponding to the whole molecules, as well as characteristic fragments. The use of APCI for the characterization of complex lipids has been reported very recently (Mutemberezi et al., 2016), and this approach is likely to gain in popularity in the current context of the development of lipidomics.

UHPLC-HRMS has led to the characterization of two families of glycerides derived, respectively, from EPA and AA, which had not been formally identified in Oomycetes to date. Furthermore, occurrence of TAG and TEPG is poorly documented and very few natural sources of these triglycerides have been described. TAG has been reported in the red algae *Gracilaria verrucosa* (Kinoshita et al., 1986). Several organisms such as *Mortierella* sp. (Shimizu et al., 1988) and the Oomycetes *Pythium* sp. (Gandhi and Weete, 1991; O'Brien et al., 1993) and *Saprolegnia* sp. (Shirasaka and Shimizu, 1995) have been reported to produce EPA-containing triglycerides. However, to our knowledge, TEPG has not been formally identified in these organisms.

In addition to EPA and AA derivatives, sporangia of *P. viticola* contain a family of C16:1 ceramides including Cer(d16:1/16:0), whose identification could be confirmed using a commercial standard. Although we could not identify them formally due to the lack of standards, other major C16:1 ceramides of *P. viticola* are very likely to include Cer(d16:1/18:0), Cer(d16:1/20:0), and Cer(d16:1/22:0) (Figure 3). Several shingophospholipids containing a C16:1 sphingosine have been detected in different plant Oomycete pathogens (Lhomme et al., 1990; Kim et al., 1998; Moreau et al., 1998). However, Cer(d16:1/16:0), Cer(d16:1/18:0), Cer(d16:1/20:0), and Cer(d16:1/22:0) had never been reported in Oomycetes before this work.

None of the lipids characterized above were detected in significant amounts in healthy grapevine leaf tissues. In addition, using an array of grapevine varieties, hybrids, and *Vitis* species with different susceptibility to downy mildew, correlation between sporulation and quantification of individual lipid markers was excellent (Figure 4), thus demonstrating that, in the context of grapevine-downy mildew interaction, these lipids can be considered as *Plasmopara*-specific. Their potential use as markers of downy mildew infection was therefore investigated.

### Lipids of *P. viticola* can be used as early markers of downy mildew infection in grapevine leaves

Most of the lipids characterized in sporangia extracts could be detected at early stages of downy mildew infection, indicating that these lipids are also present in the mycelium of *P. viticola* (Figure 5). The presence of seven major *Plasmopara*-specific lipids could easily be detected 24 h post-inoculation (Figure 6), long before the first external symptoms, which usually start to appear 4 days post-inoculation on susceptible grapevine varieties (Smith et al., 2006; Unger et al., 2007; Peressotti et al., 2011). The patterns of lipid accumulation were different in susceptible and resistant grapevine varieties. The accumulation of *Plasmopara*-specific lipids was unexpectedly higher 24 h post-inoculation in *V. riparia* than in the more susceptible varieties, suggesting a rapid development of hyphae from zoospores, even on the resistant variety (Figure 6). This is consistent with previous studies showing that *P. viticola* can form primary hyphae in the context of compatible, incompatible, and even non-host interactions (Díez-Navajas et al., 2008). However, the infection process was quickly stopped presumably as the result of plant defense reactions, the amount of detectable lipids reaching a plateau 24 h post-inoculation. Lipid accumulation in the fully susceptible variety Syrah was significantly higher than in Bianca, consistent with the partial resistance of this variety (Bellin et al., 2009).

Interestingly, the pattern of lipid accumulation was modified along the infection process. At early stages, AA and EPA were rather accumulated as fatty acids, whereas at late stages, the corresponding triacylglycerols, and especially TEPG, were massively accumulated (Figure 6), suggesting that the lipid pattern may be used as an indicator of infection developmental step. Indeed, the pattern of *Plasmopara*-specific lipids observed on Syrah leaves at late infection stage (6 days post inoculation) differed substantially from that of sporangia (Figure 6), although, at this stage, leaves were covered with an intense downy sporulation. This suggests that, even at the sporulation stage, the major part of *Plasmopara*-specific lipids comes from mycelial material localized inside grapevine leaves, this material being characterized by its high TEPG content. Altogether, these results show that *Plasmopara*-specific lipids may be used to monitor the development of downy mildew throughout the infection process, in both susceptible and resistant grapevine varieties.

### Future development of mass spectrometry-based methods for monitoring early steps of grapevine-downy mildew interaction

In this work, we have used a non-targeted metabolomic approach to characterize unusual *P. viticola*-specific lipids. Although AA, EPA, and shingophospholipids have been found in Oomycetes before (Bostock et al., 1981; Lhomme et al., 1990; Pivot et al., 1994; Judelson, 2017), the major triglycerides TAG and TEPG and the ceramide family characterized in this work had not been unambiguously identified in Oomycetes. Beyond the identification of new lipids from *P. viticola*, we show that some of these compounds can be used to efficiently monitor the infection process of downy mildew, long before the appearance of any external symptom of the disease. The availability of reliable methods for analysis of early steps of pathogen development contributes to a better understanding of plant–pathogen interactions. Genetic analysis of quantitative resistance to plant pathogens require methods allowing a precise quantification of pathogen development for a reliable detection of the genomic regions involved in resistance. Assessment of resistance to grapevine downy mildew has been traditionally performed by visually scoring disease symptoms or by measuring sporulation using a cell counter (Bellin et al., 2009). More recently, high-throughput imaging-based methods to quantify sporulation have been developed (Peressotti et al., 2011; Divilov et al., 2017). Although such high-throughput methods are very useful to screen breeding populations, they do not allow monitoring of disease progression. Pathogen growth can be monitored by quantitative PCR-based methods (Brouwer et al., 2003; Anderson and McDowell, 2015). These methods are sensitive, however, they require costly reagents and rather complicated sample preparation. Pre-symptomatic detection of downy mildew can be achieved using chlorophyll fluorescence imaging (Cséfalvay et al., 2009), but this approach is not quantitative. Through the characterization of lipid markers of *P. viticola*, we provide targets for the development of LC-MS-based methods allowing a sensitive, continuous and quantitative monitoring of downy mildew progression inside infected leaves. These methods will be likely to been successfully applied to a wide range of grapevine genotypes, including varieties and *Vitis* species. Optimization of chromatographic conditions will allow the development of fast (5 min or less) separation methods targeting the most relevant compounds, compatible with medium to high-throughput quantification. Finally, the same strategy could be applied to other important Oomycete or fungal plant pathogens, in order to develop high-sensitivity LC-MS-based methods for quantification of pathogen growth and disease progression. These methods could be used for accurate analysis of host-pathogen interaction and for the assessment of resistance to fungicides.

## Author contributions

DM and PH: conceived the original research plans and raised the funding; RB: supervised experiments and identifications of unknown compounds; LN: performed most of the experiments; DH and CR: participated in data and statistical analyses; SW-M: supervised the experiments with downy mildew; LN, DM, PH, and RB: wrote the article with contributions of all the authors.

### Conflict of interest statement

The authors declare that the research was conducted in the absence of any commercial or financial relationships that could be construed as a potential conflict of interest.
