# Supplementary material for: Identification of Lipid Markers of Plasmopara viticola Infection in Grapevine Using a Non-targeted Metabolomic Approach
Source: Front Plant Sci. 2018 Mar 21;9:360. doi: 10.3389/fpls.2018.00360 (PMC5871909; doi:10.3389/fpls.2018.00360)
Supplement: Supplementary file 1 [file Presentation1.pdf]

Supplementary Material

Identification of lipid markers of *Plasmopara viticola* infection in grapevine using a non-targeted metabolomic approach

Lise Negrel, David Halter, Sabine Wiedemann-Merdinoglu, Camille Rustenholz, Didier Merdinoglu, Philippe Hugueney, Raymonde Baltenweck*

***Correspondence:**
Raymonde Baltenweck

raymonde.baltenweck@inra.fr

Supplementary Table S1: Characteristics of the 123 major ions (peak area > 10^6), which are at least 15 times more abundant in sporangia extracts than leaf extracts, based on XCMS automated integration. Analyses were performed on one experiment including three biological replicates of extracts of *P. viticola* sporangia and of mock-inoculated leaves (Healthy_leaves). Ions are numbered according to their retention time. For all ions, the identifier, the fold change between sporangia and leaf extracts, the *m/z* and retention time (RT) are indicated. For p-value calculation, background intensity level was set to 10000 and subtracted to every integrated value. Integrated values under the background intensity were set at 1. These corrected integrated values were log10-transformed before performing Welch's two-sample *t*-tests. TPI = Time Post-Inoculation. NA refers to inapplicable statistical tests, for which the compounds could not be detected above the background level. NS = Not significant = p-value > 0.05 ; * = 0.01 < p-value ≤ 0.05 ; ** = 0.001 < p-value ≤ 0.01 ; *** = 0.001 ≤ p-value. Differential ions with p-value ≤ 0.05 were considered for further characterization.

Ion n°	identifier	fold	m/z	RT	Healthy_ leaves1	Healthy_ leaves2	Healthy_ leaves3	Sporangia1	Sporangia2	Sporangia3	p-value	signi.
1	M337T33	Inf	337.1213	33.17	0.00E+00	0.00E+00	0.00E+00	6.85E+06	1.86E+07	1.53E+07	3.50E-04	***
2	M338T33	Inf	338.1246	33.17	0.00E+00	0.00E+00	0.00E+00	1.37E+06	3.76E+06	3.10E+06	4.45E-04	***
3	M339T33	1527	339.1184	33.17	2.30E+03	2.47E+03	3.79E+03	2.20E+06	6.03E+06	4.82E+06	4.07E-04	***
4	M359T39	53	359.2577	38.81	5.11E+04	6.47E+04	9.75E+04	2.24E+06	5.00E+06	4.01E+06	2.38E-04	***
5	M377T39	220	377.2683	38.81	6.48E+04	7.96E+04	1.11E+05	1.02E+07	2.64E+07	1.95E+07	2.38E-04	***
6	M378T39	86	378.2717	38.81	5.96E+04	4.28E+04	5.67E+04	2.49E+06	6.41E+06	4.76E+06	9.17E-04	***
7	M380T41	29	380.2873	41.13	8.26E+04	5.52E+04	7.52E+04	1.04E+06	2.88E+06	2.20E+06	2.58E-03	**
8	M379T42	29	379.2839	41.59	3.36E+05	2.45E+05	3.06E+05	4.62E+06	1.19E+07	8.97E+06	3.52E-03	**
9	M356T43	35	356.2873	42.51	4.71E+04	5.54E+04	8.44E+04	1.19E+06	3.03E+06	2.29E+06	6.66E-04	***
10	M303T44	347	303.2316	43.87	1.35E+05	1.55E+05	1.80E+05	2.81E+07	7.46E+07	6.04E+07	1.26E-03	**
11	M304T44	436	304.2350	43.87	2.50E+04	1.97E+04	3.46E+04	5.93E+06	1.58E+07	1.28E+07	8.92E-05	***
12	M305T48	68	305.2474	47.97	1.95E+05	1.59E+05	2.37E+05	7.02E+06	1.83E+07	1.48E+07	1.59E-03	**
13	M281T50	16	281.2473	50.26	3.30E+05	3.09E+05	4.05E+05	2.91E+06	7.60E+06	6.07E+06	7.34E-03	**
14	M307T53	25	307.2629	52.64	1.06E+05	9.28E+04	1.06E+05	1.35E+06	3.46E+06	2.88E+06	6.57E-03	**
15	M806T79	403	806.4726	79.16	5.90E+03	0.00E+00	1.29E+04	1.21E+06	3.29E+06	3.10E+06	4.39E-02	*
16	M661T85	38	661.4825	84.66	2.32E+05	2.99E+05	9.52E+05	9.83E+06	2.35E+07	2.24E+07	3.64E-03	**
17	M482T90	256	482.2666	90.11	9.53E+03	9.27E+03	2.65E+04	2.41E+06	2.36E+06	6.83E+06	6.61E-02	.
18	M662T92	42	662.4856	91.67	8.81E+04	1.29E+05	3.97E+05	4.56E+06	1.09E+07	1.04E+07	4.38E-03	**
19	M492T96	Inf	492.4773	96.14	0.00E+00	0.00E+00	0.00E+00	4.63E+06	1.08E+07	9.30E+06	2.71E-04	***
20	M493T96	380	493.4807	96.14	9.68E+03	6.47E+03	6.18E+03	1.56E+06	3.71E+06	3.20E+06	3.30E-04	***
21	M617T103	Inf	616.5662	103.49	0.00E+00	0.00E+00	0.00E+00	1.10E+06	2.92E+06	2.44E+06	4.33E-04	***
22	M587T108	16	587.4670	107.85	7.93E+04	6.94E+04	7.95E+05	2.90E+06	6.29E+06	6.22E+06	4.30E-02	*
23	M588T108	17	588.4705	107.85	3.00E+04	1.54E+04	3.09E+05	1.15E+06	2.54E+06	2.47E+06	6.78E-02	.
24	M621T115	110	621.4876	115.10	2.17E+04	2.73E+04	4.92E+04	2.30E+06	4.14E+06	4.37E+06	7.88E-04	***
25	M622T115	587	622.4909	115.17	9.50E+02	2.91E+03	4.26E+03	1.02E+06	1.80E+06	1.94E+06	2.06E-04	***

26	M639T115	1519	639.4981	115.17	1.25E+04	1.08E+04	1.95E+04	1.18E+07	2.72E+07	2.58E+07	2.74E-03	**
27	M642T115	17	641.5042	115.48	1.15E+05	9.56E+04	1.17E+05	1.06E+06	2.53E+06	2.14E+06	5.68E-03	**
28	M641T116	3900	640.5015	115.66	2.67E+03	1.05E+03	3.71E+03	5.27E+06	1.22E+07	1.15E+07	2.86E-04	***
29	M363T123	20	363.2890	122.92	1.01E+05	8.92E+04	9.82E+04	1.02E+06	2.39E+06	2.36E+06	7.60E-03	**
30	M666T124	48	665.5134	123.80	1.41E+05	1.31E+05	1.47E+05	3.48E+06	8.49E+06	8.10E+06	5.03E-03	**
31	M667T124	245	666.5168	123.80	1.06E+04	1.92E+04	8.34E+03	1.61E+06	3.96E+06	3.76E+06	6.81E-02	.
32	M565T125	23	564.5150	125.15	6.85E+04	5.63E+04	7.77E+05	5.02E+06	7.89E+06	7.44E+06	4.50E-02	*
33	M236T125	173	236.2371	125.15	4.41E+04	4.94E+04	4.06E+04	4.25E+06	1.01E+07	8.93E+06	1.42E-03	**
34	M254T125	29	254.2477	125.15	6.91E+04	6.66E+04	5.81E+04	1.02E+06	2.41E+06	2.15E+06	4.33E-03	**
35	M502T125	Inf	502.4980	125.15	0.00E+00	0.00E+00	0.00E+00	3.56E+06	8.12E+06	7.40E+06	2.80E-04	***
36	M504T125	141	503.5013	125.15	1.14E+04	1.19E+04	2.53E+04	1.24E+06	2.95E+06	2.66E+06	7.33E-03	**
37	M521T125	14665	520.5084	125.15	6.60E+03	7.29E+03	1.38E+04	7.51E+07	1.74E+08	1.57E+08	2.76E-02	*
38	M522T125	200	521.5117	125.15	2.99E+05	2.09E+05	2.47E+05	2.80E+07	6.47E+07	5.85E+07	7.05E-04	***
39	M523T125	109	522.5150	125.15	1.01E+05	7.25E+04	8.24E+04	5.05E+06	1.20E+07	1.08E+07	1.06E-03	**
40	M539T125	16	538.5189	125.15	9.86E+04	9.00E+04	7.88E+05	2.85E+06	6.68E+06	5.97E+06	3.47E-02	*
41	M540T125	35	539.5223	125.15	1.39E+04	1.04E+04	1.37E+05	1.00E+06	2.44E+06	2.22E+06	7.26E-02	.
42	M356T126	128	356.3156	125.61	2.02E+04	2.36E+04	2.10E+04	1.64E+06	3.53E+06	3.12E+06	6.59E-04	***
43	M284T126	22	284.2945	126.06	9.81E+04	1.08E+05	9.02E+04	1.35E+06	2.69E+06	2.36E+06	2.74E-03	**
44	M541T140	714	540.5348	140.45	5.86E+03	1.70E+03	1.69E+04	3.23E+06	7.29E+06	6.92E+06	4.98E-02	*
45	M542T140	2354	541.5381	140.45	5.68E+02	2.15E+03	0.00E+00	1.15E+06	2.69E+06	2.57E+06	3.66E-04	***
46	M642T142	144	641.5134	141.81	9.67E+04	8.82E+04	9.69E+04	7.67E+06	1.65E+07	1.63E+07	2.17E-03	**
47	M643T142	153	642.5168	141.81	4.19E+04	3.70E+04	3.92E+04	3.48E+06	7.36E+06	7.22E+06	1.59E-03	**
48	M359T142	91	359.2577	142.26	3.63E+04	4.55E+04	5.05E+04	2.56E+06	4.63E+06	4.79E+06	1.38E-04	***
49	M624T146	97	623.5586	145.89	3.59E+04	1.74E+04	5.00E+04	2.05E+06	4.21E+06	3.81E+06	3.75E-03	**
50	M623T146	49	622.5551	146.34	1.85E+05	1.05E+05	1.62E+05	4.59E+06	9.02E+06	8.44E+06	1.79E-04	***
51	M668T147	8522	667.5290	147.24	0.00E+00	6.42E+02	0.00E+00	1.04E+06	2.18E+06	2.25E+06	3.13E-04	***
52	M547T150	123	547.4718	149.51	7.17E+05	5.14E+05	4.78E+05	1.57E+08	1.35E+07	4.01E+07	2.24E-02	*
53	M548T150	122	548.4753	149.51	2.73E+05	1.99E+05	1.80E+05	5.92E+07	5.19E+06	1.48E+07	2.17E-02	*
54	M527T153	2379	526.5192	152.67	0.00E+00	7.29E+02	3.74E+03	2.36E+06	4.71E+06	3.56E+06	1.79E-04	***
55	M644T160	66	643.5292	159.58	4.10E+04	3.85E+04	5.39E+04	1.55E+06	3.68E+06	3.59E+06	1.09E-03	**
56	M619T164	216	618.5617	163.55	9.69E+03	1.93E+04	7.23E+04	5.38E+06	8.99E+06	7.50E+06	1.17E-01	NS
57	M618T164	26	617.5659	163.97	1.11E+04	5.78E+03	2.01E+05	1.44E+06	2.39E+06	1.89E+06	1.50E-01	NS
58	M557T164	Inf	556.5449	164.41	0.00E+00	0.00E+00	0.00E+00	3.80E+06	8.46E+06	6.91E+06	2.38E-04	***
59	M576T164	Inf	575.5585	164.41	0.00E+00	0.00E+00	0.00E+00	3.81E+07	8.45E+07	6.99E+07	1.80E-04	***
60	M577T164	12373	576.5620	164.41	0.00E+00	3.22E+03	0.00E+00	7.86E+06	1.75E+07	1.44E+07	2.18E-04	***
61	M558T164	2909	557.5483	164.46	0.00E+00	1.96E+03	6.98E+02	1.52E+06	3.38E+06	2.84E+06	2.75E-04	***
62	M575T164	277242	574.5553	164.46	0.00E+00	1.66E+03	0.00E+00	9.28E+07	2.02E+08	1.67E+08	1.55E-04	***
63	M578T164	108	577.5652	164.46	2.19E+04	9.66E+03	1.80E+04	1.04E+06	2.36E+06	1.98E+06	1.15E-01	NS
64	M593T164	977	592.5657	164.46	1.41E+04	1.29E+03	4.82E+03	4.01E+06	8.57E+06	7.16E+06	4.25E-02	*
65	M378T165	362	378.3364	164.51	1.70E+04	6.40E+03	3.86E+03	2.12E+06	4.16E+06	3.59E+06	5.47E-02	.
66	M236T165	272	236.2371	164.83	3.31E+04	3.91E+04	2.50E+04	5.17E+06	1.16E+07	9.66E+06	6.51E-05	***

67	M254T165	42	254.2476	164.83	6.08E+04	5.44E+04	3.38E+04	1.23E+06	2.73E+06	2.32E+06	3.09E-04	***
68	M633T165	74	632.5775	164.90	1.71E+04	1.16E+04	1.53E+05	3.54E+06	5.27E+06	4.58E+06	4.53E-02	*
69	M550T165	32	549.5432	165.39	5.54E+05	6.46E+05	1.11E+06	1.35E+07	3.21E+07	2.83E+07	7.16E-04	***
70	M531T166	4670	530.5294	165.69	0.00E+00	1.74E+03	0.00E+00	1.42E+06	3.60E+06	3.09E+06	3.86E-04	***
71	M549T166	1134	548.5399	165.69	6.94E+04	5.85E+04	3.77E+04	3.44E+07	8.13E+07	7.22E+07	4.14E-05	***
72	M551T166	16	550.5466	165.69	2.09E+05	2.40E+05	4.22E+05	2.57E+06	6.16E+06	5.44E+06	1.60E-03	**
73	M567T166	386	566.5502	165.80	5.65E+03	2.13E+03	9.88E+03	1.27E+06	2.93E+06	2.61E+06	3.26E-04	***
74	M549T171	18	549.4876	171.48	6.65E+05	7.49E+05	1.25E+06	1.86E+07	1.41E+07	1.45E+07	1.27E-03	**
75	M550T172	15	550.4909	171.84	3.34E+05	3.68E+05	6.39E+05	9.38E+06	5.47E+06	5.64E+06	5.96E-04	***
76	M583T182	Inf	582.5606	182.37	0.00E+00	0.00E+00	0.00E+00	1.25E+06	2.81E+06	2.25E+06	2.81E-04	***
77	M602T182	Inf	601.5743	182.37	0.00E+00	0.00E+00	0.00E+00	1.36E+07	3.01E+07	2.45E+07	1.99E-04	***
78	M603T182	Inf	602.5779	182.37	0.00E+00	0.00E+00	0.00E+00	2.85E+06	6.50E+06	5.20E+06	2.59E-04	***
79	M619T182	56	618.5815	182.37	1.73E+04	2.95E+04	7.95E+04	1.43E+06	3.13E+06	2.55E+06	1.16E-02	*
80	M601T183	73375	600.5710	182.52	4.65E+02	7.35E+02	9.43E+02	3.18E+07	6.95E+07	5.60E+07	1.73E-04	***
81	M646T183	56	645.5811	182.70	2.12E+04	2.13E+03	9.64E+04	1.44E+06	2.92E+06	2.29E+06	1.59E-01	NS
82	M645T183	Inf	644.5777	182.80	0.00E+00	0.00E+00	0.00E+00	3.18E+06	6.18E+06	5.31E+06	1.72E-04	***
83	M563T192	573	562.5557	191.72	5.21E+03	4.00E+03	4.00E+03	1.32E+06	3.31E+06	2.94E+06	3.91E-04	***
84	M659T193	326	658.5934	192.70	1.09E+03	4.76E+02	2.07E+04	1.60E+06	3.09E+06	2.56E+06	6.41E-02	.
85	M589T198	574	588.5712	197.64	8.65E+03	0.00E+00	0.00E+00	1.17E+06	2.09E+06	1.70E+06	1.43E-04	***
86	M616T202	1352	615.5901	202.01	4.19E+03	1.15E+03	7.37E+02	1.72E+06	3.60E+06	2.89E+06	2.21E-04	***
87	M615T202	3739	614.5866	202.19	0.00E+00	2.86E+03	2.10E+03	3.96E+06	8.09E+06	6.50E+06	1.84E-04	***
88	M633T202	468	632.5972	202.19	2.78E+04	1.82E+04	1.49E+05	1.97E+07	3.92E+07	3.23E+07	1.09E-02	*
89	M634T202	27	633.6004	202.19	5.76E+05	4.23E+05	5.52E+05	8.89E+06	1.77E+07	1.46E+07	9.14E-04	***
90	M674T202	25	673.6124	202.44	1.05E+05	5.68E+04	9.05E+04	1.53E+06	2.57E+06	2.09E+06	3.96E-04	***
91	M673T202	1598	672.6090	202.50	5.78E+02	1.23E+03	6.63E+03	3.42E+06	5.53E+06	4.53E+06	8.34E-05	***
92	M621T223	72	620.5781	223.16	5.04E+04	2.97E+04	1.81E+05	4.40E+06	7.53E+06	6.79E+06	1.29E-02	*
93	M236T223	81	236.2372	223.18	5.81E+04	5.70E+04	4.76E+04	2.42E+06	5.64E+06	5.08E+06	1.83E-03	**
94	M412T223	38	412.3784	223.18	4.50E+04	4.34E+04	7.29E+04	1.22E+06	2.56E+06	2.32E+06	2.76E-04	***
95	M577T223	54288	576.5713	223.18	2.59E+03	3.58E+03	0.00E+00	6.09E+07	1.42E+08	1.32E+08	2.15E-04	***
96	M579T223	1557	578.5779	223.18	8.17E+03	4.01E+03	5.96E+03	5.15E+06	1.20E+07	1.11E+07	2.86E-04	***
97	M595T223	26	594.5818	223.18	7.58E+04	2.32E+04	4.29E+05	2.51E+06	5.72E+06	5.33E+06	4.71E-02	*
98	M559T224	Inf	558.5609	223.62	0.00E+00	0.00E+00	0.00E+00	2.63E+06	5.83E+06	5.43E+06	2.78E-04	***
99	M578T224	1660	577.5747	223.68	4.39E+04	3.31E+04	7.10E+03	2.53E+07	5.93E+07	5.51E+07	8.67E-02	.
100	M603T230	Inf	602.5871	230.05	0.00E+00	0.00E+00	0.00E+00	5.25E+07	1.04E+08	8.64E+07	1.26E-04	***
101	M585T230	Inf	584.5765	230.10	0.00E+00	0.00E+00	0.00E+00	2.03E+06	4.08E+06	3.53E+06	2.04E-04	***
102	M605T230	Inf	604.5936	230.10	0.00E+00	0.00E+00	0.00E+00	5.00E+06	1.00E+07	8.27E+06	1.71E-04	***
103	M621T231	47	620.5971	230.57	3.67E+04	7.48E+03	1.37E+05	1.82E+06	3.92E+06	2.84E+06	1.78E-01	NS
104	M604T231	9463	603.5903	230.70	6.18E+03	5.12E+03	0.00E+00	2.30E+07	4.59E+07	3.80E+07	1.41E-04	***
105	M646T231	30	645.5970	230.90	3.78E+04	5.83E+03	1.17E+05	1.21E+06	1.94E+06	1.62E+06	1.96E-01	NS
106	M647T231	228	646.5934	230.90	2.15E+04	1.68E+04	4.42E+04	4.70E+06	7.55E+06	6.60E+06	3.48E-03	**

107	M661T231	48	660.6092	231.03	5.87E+04	3.78E+04	1.18E+05	2.99E+06	4.12E+06	3.25E+06	6.27E-03	**
108	M615T238	333	614.5867	238.07	4.34E+04	8.17E+04	0.00E+00	4.09E+06	1.92E+07	1.84E+07	1.26E-01	NS
109	M629T248	226	628.6025	247.79	4.29E+03	1.27E+04	1.55E+04	1.47E+06	3.09E+06	2.77E+06	7.92E-02	.
110	M597T255	1777	596.5977	255.47	5.75E+02	0.00E+00	3.15E+03	1.21E+06	2.76E+06	2.64E+06	3.43E-04	***
111	M631T314	132	630.6182	314.47	1.09E+04	7.44E+03	5.99E+04	2.15E+06	4.47E+06	3.75E+06	1.01E-01	NS
112	M949T473	Inf	948.7059	472.85	0.00E+00	0.00E+00	0.00E+00	1.69E+06	3.88E+06	4.11E+06	3.74E-04	***
113	M359T473	105	359.2580	473.32	5.06E+04	4.53E+04	4.47E+04	2.43E+06	5.98E+06	6.29E+06	3.42E-03	**
114	M644T473	6105	644.4754	473.32	6.10E+03	2.70E+03	4.74E+03	1.41E+07	3.52E+07	3.34E+07	3.03E-04	***
115	M645T473	111	645.4759	473.32	4.22E+04	3.52E+04	5.26E+04	2.49E+06	5.95E+06	5.99E+06	6.47E-04	***
116	M643T474	1799	643.4721	473.78	3.84E+04	2.42E+04	3.92E+04	3.09E+07	7.98E+07	7.24E+07	5.22E-05	***
117	M946T474	Inf	945.6964	473.78	0.00E+00	0.00E+00	0.00E+00	2.92E+07	7.49E+07	6.95E+07	2.89E-04	***
118	M947T474	Inf	946.6995	473.78	0.00E+00	0.00E+00	0.00E+00	2.00E+07	5.16E+07	4.75E+07	3.03E-04	***
119	M948T474	11923	947.7031	473.78	0.00E+00	1.48E+03	2.01E+03	6.99E+06	1.80E+07	1.67E+07	3.45E-04	***
120	M620T519	159	620.4748	519.47	1.85E+04	2.17E+04	1.87E+04	1.00E+06	4.05E+06	4.32E+06	5.49E-03	**
121	M619T520	75	619.4715	519.94	1.19E+05	9.66E+04	1.16E+05	4.46E+06	1.01E+07	1.03E+07	2.45E-03	**
122	M923T520	Inf	922.6989	519.94	0.00E+00	0.00E+00	0.00E+00	3.61E+06	8.36E+06	8.43E+06	3.24E-04	***
123	M922T520	Inf	921.6957	519.95	0.00E+00	0.00E+00	0.00E+00	2.92E+06	1.24E+07	1.29E+07	9.51E-04	***

Supplementary Table S2: List of the *Plasmopara*-specific ions identified by non-targeted metabolomics, which have been characterized in this work. Ions are numbered according to their retention time and are organized as derivatives of EPA, AA and ceramides, indicated in green, blue and red, respectively. Major compounds are indicated in bold. Identifications confirmed by the corresponding standards are marked by a grey background. Putative identifications are indicated with italics. For all ions, the identifier, the *m/z*, retention time (RT) and fold change between sporangia and leaf extracts are indicated.

Ion n°	Identifier	Fold	*m/z*	RT (s)	Ion formula	*(Putative)* identification
**10**	**M303T44**	**347**	**303,2316**	**43,87**	**$C_{20}H_{30}O_2$**	**eicosapentaenoic acid (EPA)**
11	M304T44	436	304,2350	43,87		isotope of M303T44
**5**	**M377T39**	**220**	**377,2683**	**38,81**	**$C_{23}H_{37}O_4$**	**eicosapentaenoyl-glycerol (EPG)**
6	M378T39	86	378,2717	38,81		isotope of M377T39
4	M359T39	53	359,2577	38,81	$C_{23}H_{35}O_3$	fragment EPG $[M-H_2O+H]^+$
**16**	**M661T85**	**38**	**661,4825**	**84,66**		**dieicosapentaenoyl-glycerol (DEPG)**
18	M662T92	42	662,4856	91,67		isotope of M661T85
**117**	**M946T474**	**Inf**	**945,6964**	**473,78**	**$C_{63}H_{93}O_6$**	**trieicosapentaenoyl-glycerol (TEPG)**
118	M947T474	Inf	946,6995	473,78		isotope of M946T474
119	M948T474	11923	947,7031	473,78		isotope of M946T474
112	M949T473	Inf	948,7059	472,85		isotope of M946T474
113	M359T473	105	359,2580	473,32	$C_{23}H_{35}O_3$	fragment EPG
116	M643T474	1799	643,4721	473,78	$C_{46}H_{59}O_2$	fragment EPA-diglyceryl
114	M644T473	6105	644,4754	473,32		isotope of M643T473
115	M645T473	111	645,4759	473,32		isotope of M643T473
**12**	**M305T48**	**68**	**305,2474**	**47,97**	**$C_{20}H_{32}O_2$**	**arachidonic acid (AA)**
**8**	**M379T42**	**29**	**379,2839**	**41,59**	**$C_{23}H_{39}O_4$**	**arachidonoyl-glycerol (AG)**
7	M380T41	29	380,2873	41,13		isotope of M379T42
**30**	**M666T124**	**48**	**665,5134**	**123,80**	**$C_{43}H_{69}O_5$**	**diarachidonoyl-glycerol (DAG)**
31	M667T124	245	666,5168	123,80		isotope of M666T124
**19**	**M492T96**	**Inf**	**492,4773**	**96,14**	**$C_{32}H_{64}NO_3$**	**Cer(d16:1/16:0)**
20	M493T96	380	493,4807	96,14		isotope of M492T96
**40**	**M539T125**	**16**	**538,5189**	**125,15**	**$C_{34}H_{68}NO_3$**	***Cer(d16:1/18:0)***
41	M540T125	35	539,5223	125,15		*isotope of M539T125*
37	M521T125	14665	520,5084	125,15	$C_{34}H_{66}NO_2$	*fragment* $[M-H_2O+H]^+$
38	M522T125	200	521,5117	125,15		*isotope of M521T125*
39	M523T125	109	522,5150	125,15		*isotope of M521T125*
35	M502T125	Inf	502,4980	125,15	$C_{34}H_{64}NO$	*fragment* $[M-2H_2O+H]^+$
36	M504T125	141	503,5013	125,15		*isotope of M502T125*
33	M236T125	173	236,2371	125,15	$C_{16}H_{30}N$	*fragment N from Cer(d16:1/18:0)*
34	M254T125	29	254,2477	125,15	$C_{16}H_{32}NO$	*fragment N' from Cer(d16:1/18:0)*
42	M356T126	128	356,3156	125,61	$C_{21}H_{42}NO_3$	*fragment C from Cer(d16:1/18:0)*
43	M284T126	22	284,2945	126,06	$C_{18}H_{38}NO$	*fragment A from Cer(d16:1/18:0)*
**73**	**M567T166**	**386**	**566,5502**	**165,80**	**$C_{36}H_{72}NO_3$**	***Cer(d16:1/20:0)***
71	M549T166	1134	548,5399	165,69	$C_{36}H_{70}NO_2$	*fragment* $[M-H_2O+H]^+$
69	M550T165	32	549,5432	165,39		*isotope of M549T166*
72	M551T166	16	550,5466	165,69		*isotope of M549T166*
70	M531T166	4670	530,5294	165,69	$C_{36}H_{68}NO$	*fragment* $[M-2H_2O+H]^+$
66	M236T165	272	236,2371	164,83	$C_{16}H_{30}N$	*fragment N from Cer(d16:1/20:0)*
67	M254T165	42	254,2476	164,83	$C_{16}H_{32}NO$	*fragment N' from Cer(d16:1/20:0)*
**97**	**M595T223**	**26**	**594,5818**	**223,18**	**$C_{38}H_{76}NO_3$**	***Cer(d16:1/22:0)***
95	M577T223	54288	576,5713	223,18	$C_{38}H_{72}NO_2$	*fragment* $[M-H_2O+H]^+$
99	M578T224	1660	577,5747	223,68		*isotope of M577T223*
96	M579T223	1557	578,5779	223,18		*isotope of M577T223*
98	M559T224	Inf	558,5609	223,62	$C_{38}H_{70}NO$	*fragment* $[M-2H_2O+H]^+$
93	M236T223	81	236,2372	223,18	$C_{16}H_{30}N$	*fragment N from Cer(d16:1/22:0)*
94	M412T223	38	412,3784	223,18	$C_{25}H_{50}NO_3$	*fragment C from Cer(d16:1/22:0)*
13	M281T50	16	281,2473	50,26	**$C_{18}H_{32}O_2$**	*$C_{18:2}$ acid*
**46**	**M642T142**	**144**	**641,51342**	**141,805**	**$C_{41}H_{69}O_5$**	***diglyceride 20:5/18:1***
47	M643T142	153	642,51677	141,805		*isotope of M642T142*
48	M359T142	91	359,2577	142,260	$C_{23}H_{35}O_3$	*fragment EPG*

Supplementary Table S3: APCI(+)MS data for ceramides from *P. viticola* (n° **19**, **40**, **73**, **97**) and for the commercial ceramide standards Cer(d16:1/16:0) and Cer(d18:1/20:0). For fragments nomenclature, see Figure S1. The most abundant fragments in each spectrum is indicated in bold. Fragments listed in XCMS table are in italics. For all ions, error in mass accuracy is under 1 ppm.

**compound**	***19***	***40***	***73***	***97***	***Cer(d16:1/16:0)***	***Cer(d18:1/20:0)***
**structure**	***Cer(d16:1/16:0)***	***Cer(d16:1/18:0)***	***Cer(d16:1/20:0)***	***Cer(d16:1/22:0)***		
**RT (min)**	1.6	2.08	2.74	3.72	1.6	4.20
**Chemical formula**	$C_{32}H_{63}O_3N$	$C_{34}H_{67}O_3N$	$C_{36}H_{71}O_3N$	*$C_{38}H_{75}O_3N$*	$C_{32}H_{63}O_3N$	*$C_{38}H_{75}O_3N$*
**fragments**						
[M + H]+	510.4877	*538.5189*	*566.5502*	*594.5818*	510.4877	594.5819
[M-H_2O+H]+	***492.4773***	***520.5084***	***548.5399***	***576.5713***	**492.4772**	**576.5714**
[M-2H_2O+H]+	474.4667	*502.4979*	*530.5294*	*558.5609*	474.4667	558.561
[M-H_2O-CH_2O+H]+	462.4665	490.4981	518.5301	546.5604	462.4665	546.5615
Sphingosine						
fragment **N**	236.2371	*236.2371*	*236.2371*	*236.2372*	236.2372	264.2683
fragment **N'**	254.2476	*254.2476*	*254.2476*	254.2476	254.2476	282.2791
Fatty acid						
fragment **A**	256.2635	*284.2945*	312.3257	340.3574	256.2631	312.326
fragment **B**	298.2754	326.3051	354.3357	382.3681	298.2751	354.3366
fragment **C**	328.2844	*356.3156*	384.3467	*412.3784*	328.2846	384.3474

Supplementary Table S4: P-values and significances of Welch's *t*-test comparing inoculated and non-inoculated samples for each variety and for each compound, at each time point. Genotypes with different susceptibility to downy mildew were used: Syrah (fully susceptible), Bianca (partially resistant) and *V. riparia* (resistant). For each variety, inoculated (I) and non-inoculated (NI) leaf discs were analyzed for *Plasmopara*-specific lipids 0h, 24h, 48h, 72h and 144h post treatment. Three biological replicates were analyzed for each of these 30 conditions, representing a total of 90 analyzes. For p-value calculation, background intensity level was set to 10000 and subtracted to every integrated value. Integrated values under the background intensity were set at 1. These corrected integrated values were log10-transformed before performing Welch's two-sample *t*-tests. TPI = Time Post-Inoculation. NA refers to inapplicable statistical tests, for which the compounds could not be detected above the background level. NS = Not significant = p-value > 0.05 ; * = 0.01 < p-value ≤ 0.05 ; ** = 0.001 < p-value ≤ 0.01 ; *** = 0.001 ≤ p-value.

Variety	TPI	AA		AG		DAG		EPA		EPG		DEPG		TEPG		Cer(d16:1/16:0)		Cer(d16:1/18:0)		Cer(d16:1/20:0)		Cer(d16:1/22:0)	
		Pvalue	Signi	Pvalue	Signi	Pvalue	Signi	Pvalue	Signi	Pvalue	Signi	Pvalue	Signi	Pvalue	Signi	Pvalue	Signi	Pvalue	Signi	Pvalue	Signi	Pvalue	Signi
Syrah	0h	NA	NS	NA	NS	NA	NS	NA	NS	NA	NS	NA	NS	NA	NS	NA	NS	NA	NS	NA	NS	NA	NS
Syrah	24h	NA	NS	NA	NS	NA	NS	7,09E-04	***	NA	NS	5,46E-04	***	4,23E-01	NS	5,96E-04	***	5,17E-04	***	3,69E-04	***	2,50E-04	***
Syrah	48h	NA	NS	NA	NS	4,23E-01	NS	2,76E-04	***	NA	NS	3,51E-04	***	1,84E-01	NS	1,59E-03	**	8,62E-04	***	4,28E-04	***	2,77E-04	***
Syrah	72h	2,21E-03	**	NA	NS	4,23E-01	NS	1,09E-03	**	NA	NS	5,25E-04	***	9,77E-04	***	2,72E-03	**	5,43E-04	***	3,35E-04	***	3,23E-04	***
Syrah	6dpi	5,18E-04	***	2,88E-04	***	3,39E-04	***	4,05E-04	***	1,25E-04	***	2,56E-04	***	1,68E-05	***	1,48E-04	***	7,09E-05	***	2,16E-04	***	1,02E-04	***
Bianca	0h	NA	NS	NA	NS	NA	NS	NA	NS	NA	NS	NA	NS	NA	NS	NA	NS	NA	NS	NA	NS	NA	NS
Bianca	24h	NA	NS	NA	NS	NA	NS	1,94E-02	*	NA	NS	1,84E-01	NS	NA	NS	1,98E-01	NS	4,23E-01	NS	1,05E-02	*	1,67E-03	**
Bianca	48h	NA	NS	NA	NS	NA	NS	2,92E-04	***	NA	NS	2,24E-03	**	1,86E-01	NS	4,23E-01	NS	1,84E-01	NS	8,05E-04	***	7,36E-04	***
Bianca	72h	NA	NS	NA	NS	NA	NS	1,95E-03	**	NA	NS	1,98E-03	**	9,41E-04	***	NA	NS	1,42E-02	*	7,78E-04	***	7,14E-04	***
Bianca	6dpi	1,01E-04	***	NA	NS	1,84E-01	NS	1,86E-05	***	3,35E-04	***	2,28E-04	***	1,67E-04	***	1,56E-03	**	2,58E-04	***	2,01E-04	***	1,89E-04	***
V. riparia	0h	NA	NS	NA	NS	NA	NS	NA	NS	NA	NS	NA	NS	NA	NS	NA	NS	NA	NS	NA	NS	NA	NS
V. riparia	24h	4,62E-04	***	NA	NS	NA	NS	9,62E-05	***	NA	NS	4,38E-04	***	4,23E-01	NS	4,77E-04	***	5,60E-04	***	3,65E-04	***	1,66E-04	***
V. riparia	48h	NA	NS	NA	NS	NA	NS	1,16E-03	**	NA	NS	1,21E-04	***	8,76E-03	**	3,38E-04	***	8,86E-05	***	4,69E-04	***	8,23E-05	***
V. riparia	72h	NA	NS	NA	NS	NA	NS	9,54E-04	***	NA	NS	1,20E-04	***	2,47E-03	**	1,47E-03	**	2,97E-03	**	6,59E-04	***	5,06E-04	***
V. riparia	6dpi	NA	NS	NA	NS	NA	NS	4,23E-01	NS	NA	NS	7,44E-04	***	1,84E-01	NS	1,84E-01	NS	8,13E-05	***	4,82E-04	***	3,57E-05	***

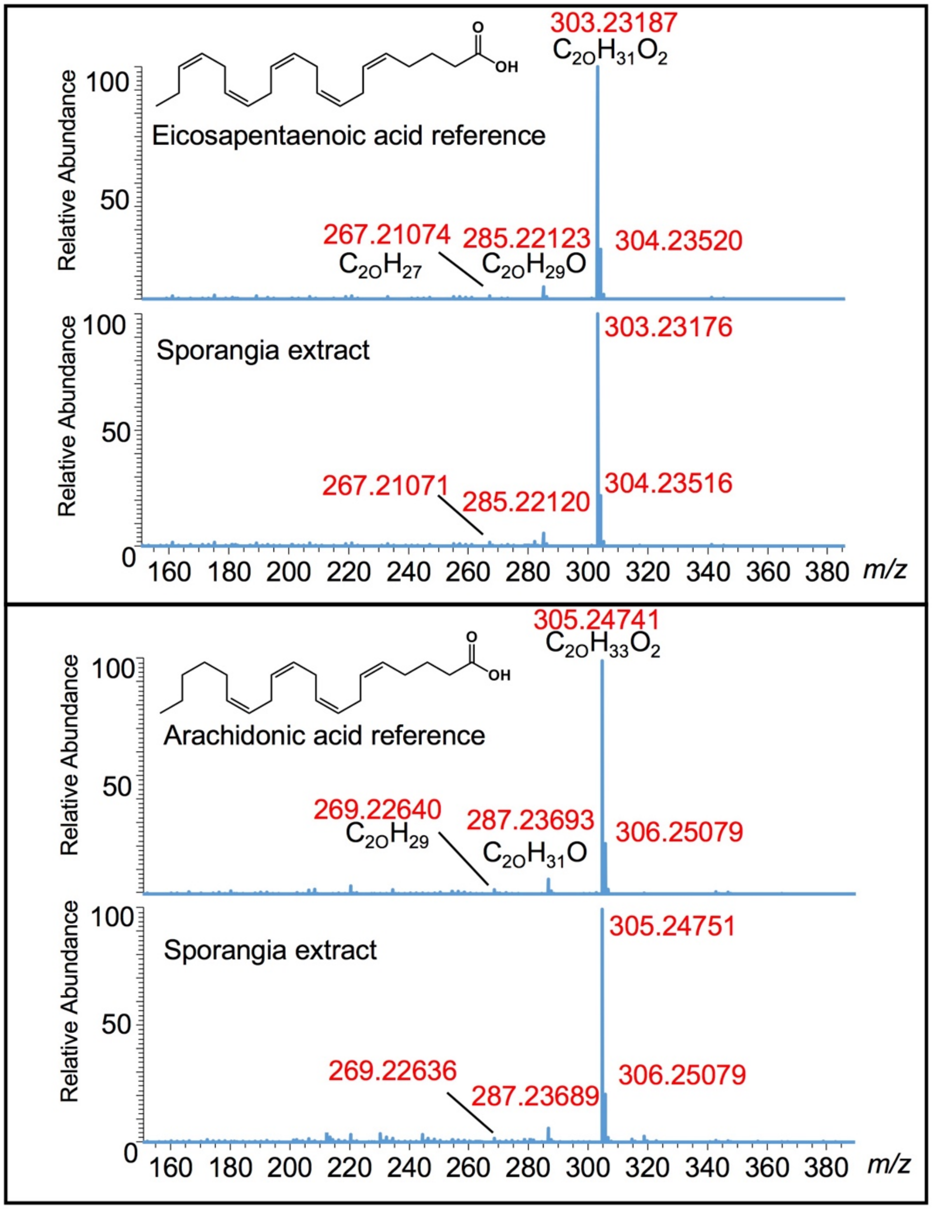

Supplementary Figure S1: Identification of EPA and AA in *P. viticola* sporangia extract by comparison of their mass spectrum to that of the corresponding commercial reference. Spectra were obtained with an APCI source operating in positive mode.

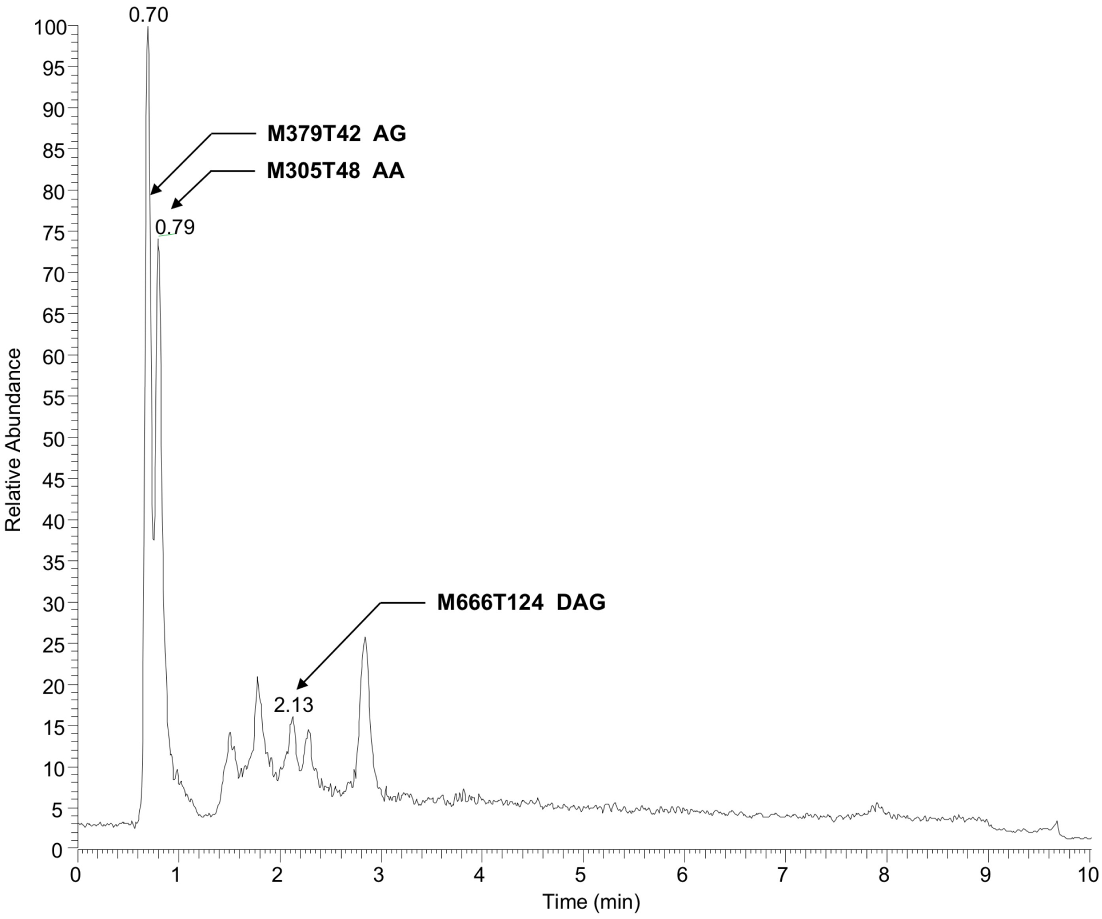

Supplementary Figure S2: EIC of sporangia extracts for *m/z* 287.2369 (± 2 ppm), a characteristic ion of AA. Peaks corresponding to *Plasmopara*-specific ions identified by the non-targeted metabolomic approach (Table 1) are indicated. Compounds whose identity has been confirmed by using the corresponding standards are indicated in bold.

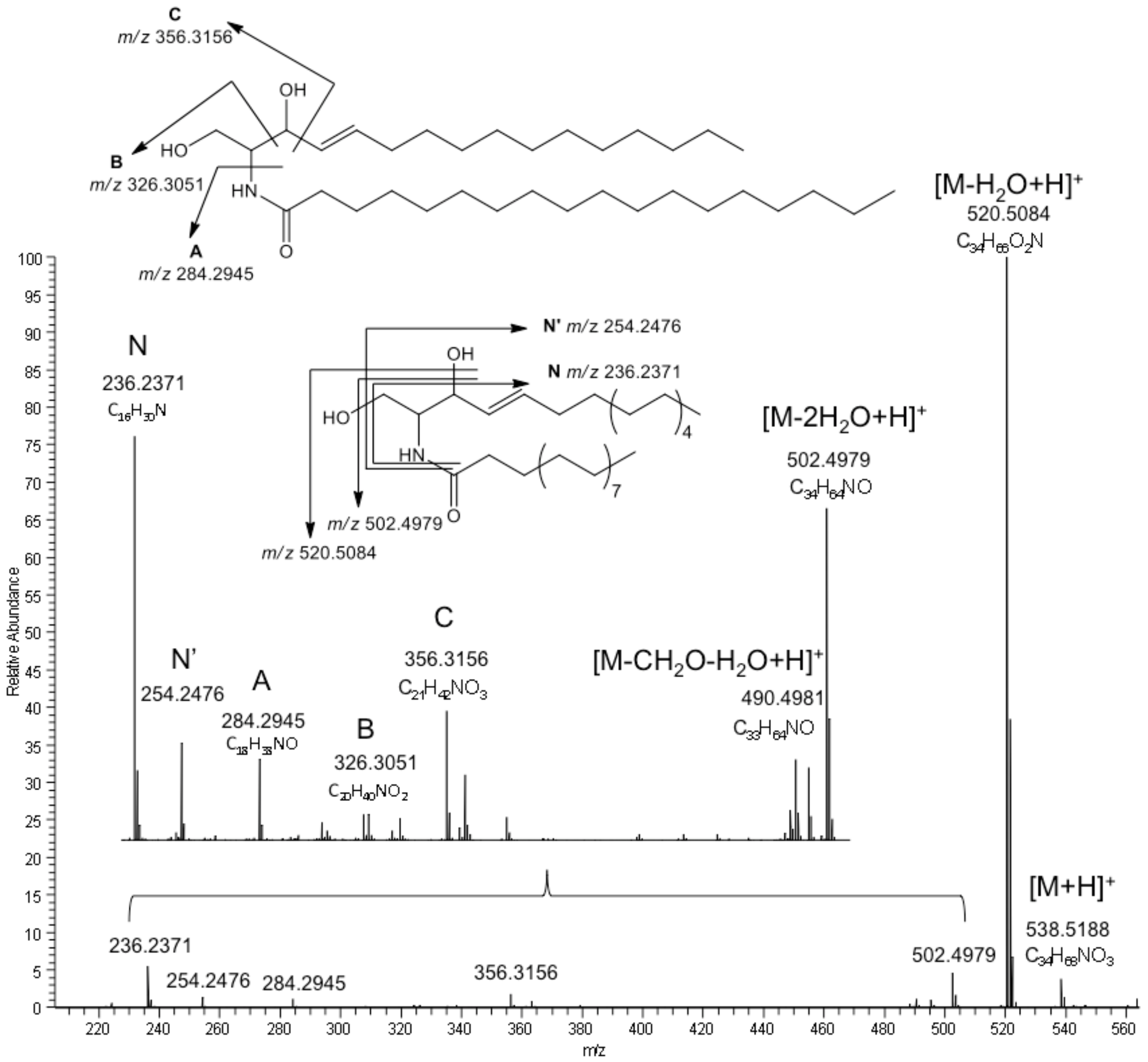

Supplementary Figure S3: Mass spectrum of putative Cer(d16:1/18:0) (M521T125, n°37) from *P. viticola.* The fragmentation giving rise to the main fragments is indicated.

Supplementary Figure S4: Kinetic analysis of *Plasmopara*-specific lipids accumulation following downy mildew infection in different grapevine cultivars. Grapevine cultivars with different susceptibility to downy mildew were used: Syrah (fully susceptible), Bianca (partially resistant) and *V. riparia* (resistant). For each variety, inoculated (I) and non-inoculated (NI) leaf discs were analyzed for *Plasmopara*-specific lipids 0h, 24h, 48h, 72h and 144h post treatment. Three biological replicates were analyzed for each of these 30 conditions, representing a total of 90 analyses. Means are presented, with bars indicating standard deviation.

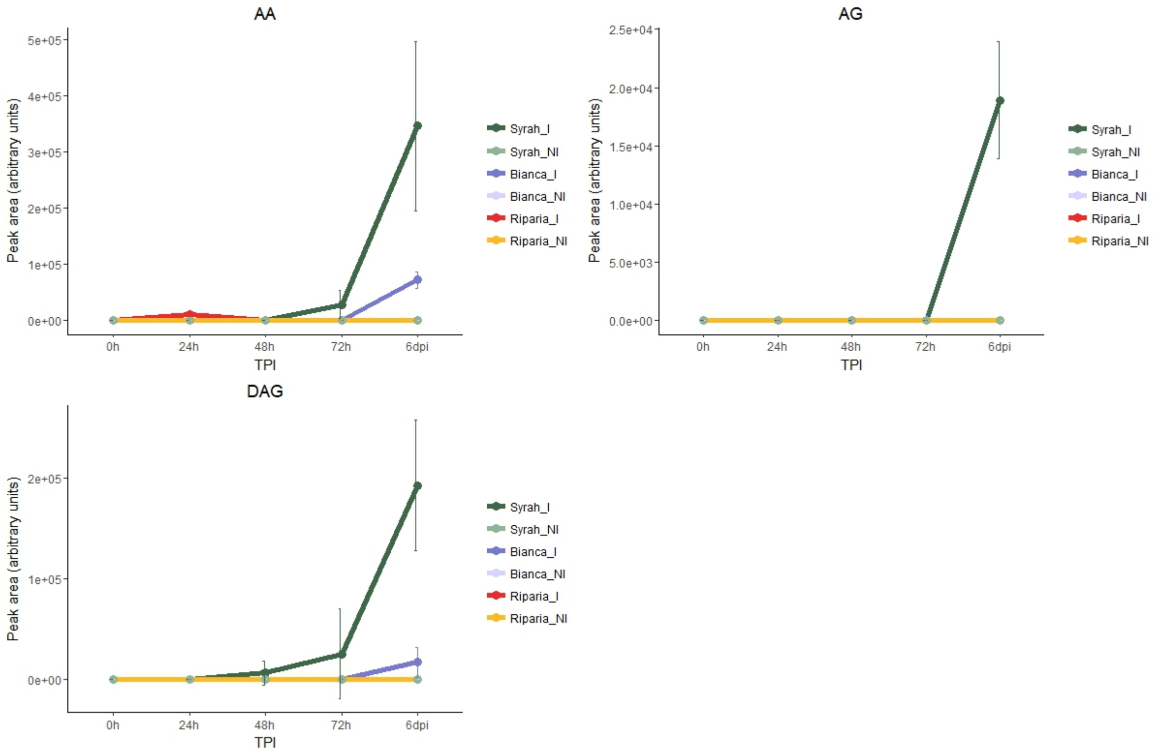

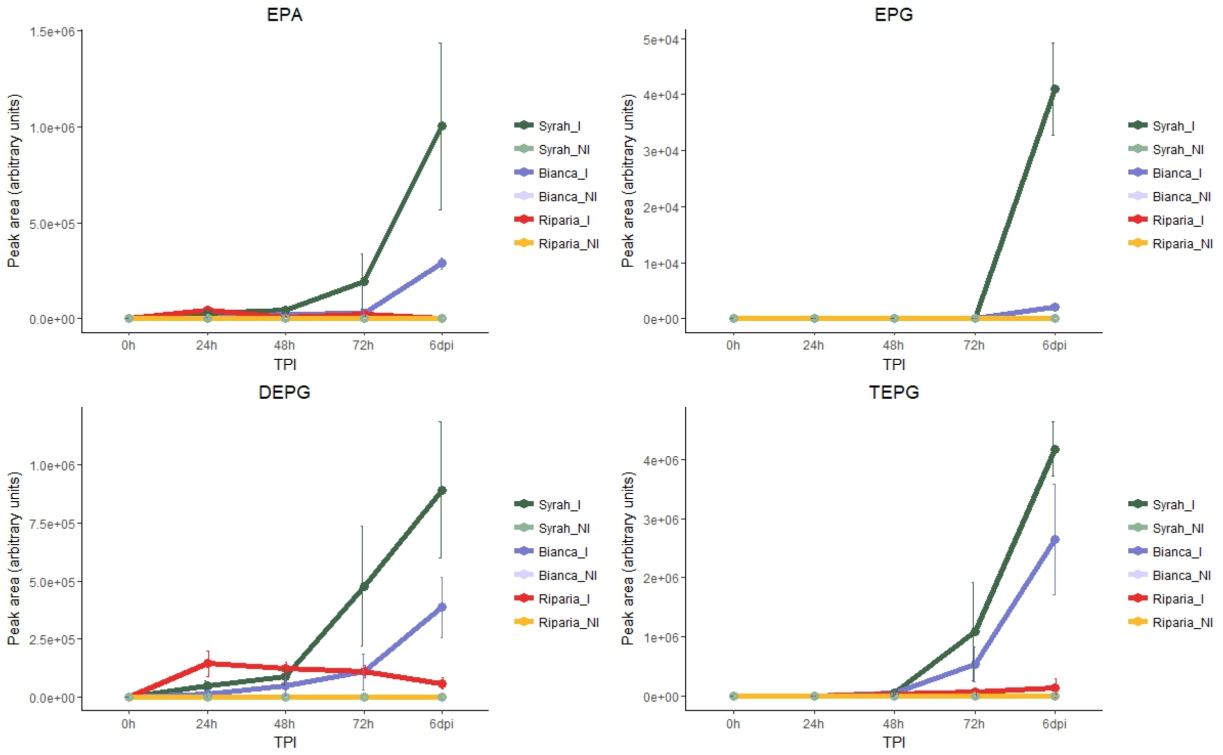
EPA
Peak area (arbitrary units)
1.5e+06
1.0e+06
5.0e+05
0.0e+00
0h
24h
48h
72h
6dpi
TPI
Syrah_I
Syrah_NI
Bianca_I
Bianca_NI
Riparia_I
Riparia_NI
EPG
Peak area (arbitrary units)
5e+04
4e+04
3e+04
2e+04
1e+04
0e+00
0h
24h
48h
72h
6dpi
TPI
Syrah_I
Syrah_NI
Bianca_I
Bianca_NI
Riparia_I
Riparia_NI
DEPG
Peak area (arbitrary units)
1.0e+06
7.5e+05
5.0e+05
2.5e+05
0.0e+00
0h
24h
48h
72h
6dpi
TPI
Syrah_I
Syrah_NI
Bianca_I
Bianca_NI
Riparia_I
Riparia_NI
TEPG
Peak area (arbitrary units)
4e+06
3e+06
2e+06
1e+06
0e+00
0h
24h
48h
72h
6dpi
TPI
Syrah_I
Syrah_NI
Bianca_I
Bianca_NI
Riparia_I
Riparia_NI

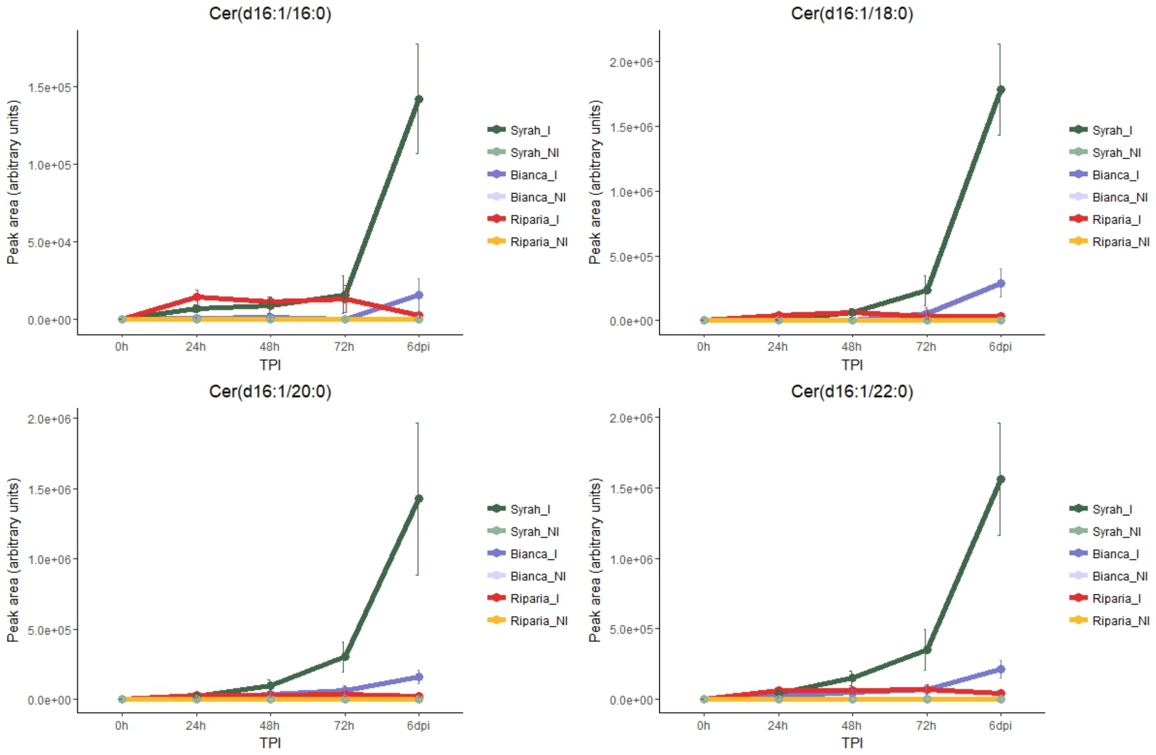
Cer(d16:1/16:0)
Peak area (arbitrary units)
1.5e+05
1.0e+05
5.0e+04
0.0e+00
0h
24h
48h
72h
6dpi
TPI
Syrah_I
Syrah_NI
Bianca_I
Bianca_NI
Riparia_I
Riparia_NI
Cer(d16:1/18:0)
Peak area (arbitrary units)
2.0e+06
1.5e+06
1.0e+06
5.0e+05
0.0e+00
0h
24h
48h
72h
6dpi
TPI
Syrah_I
Syrah_NI
Bianca_I
Bianca_NI
Riparia_I
Riparia_NI
Cer(d16:1/20:0)
Peak area (arbitrary units)
2.0e+06
1.5e+06
1.0e+06
5.0e+05
0.0e+00
0h
24h
48h
72h
6dpi
TPI
Syrah_I
Syrah_NI
Bianca_I
Bianca_NI
Riparia_I
Riparia_NI
Cer(d16:1/22:0)
Peak area (arbitrary units)
2.0e+06
1.5e+06
1.0e+06
5.0e+05
0.0e+00
0h
24h
48h
72h
6dpi
TPI
Syrah_I
Syrah_NI
Bianca_I
Bianca_NI
Riparia_I
Riparia_NI
